# Single-nucleus RNA-seq reveals that MBD5, MBD6, and SILENZIO maintain silencing in the vegetative cell of developing pollen

**DOI:** 10.1016/j.celrep.2022.111699

**Published:** 2022-11-22

**Authors:** Lucia Ichino, Colette L. Picard, Jaewon Yun, Meera Chotai, Shuya Wang, Evan K. Lin, Ranjith K. Papareddy, Yan Xue, Steven E. Jacobsen

**Affiliations:** 1 Molecular Biology Institute, University of California Los Angeles, Los Angeles, CA 90095, USA; 2 Department of Molecular, Cell and Developmental Biology, University of California Los Angeles, Los Angeles, CA 90095, USA; 3 Eli and Edyth Broad Center of Regenerative Medicine and Stem Cell Research, University of California Los Angeles, Los Angeles, CA 90095, USA; 4 Howard Hughes Medical Institute (HHMI), UCLA, Los Angeles, CA 90095, USA; 5 Lead contact

## Abstract

Silencing of transposable elements (TEs) drives the evolution of numerous redundant mechanisms of transcriptional regulation. *Arabidopsis* MBD5, MBD6, and SILENZIO act as TE repressors downstream of DNA methylation. Here, we show, via single-nucleus RNA-seq of developing male gametophytes, that these repressors are critical for TE silencing in the pollen vegetative cell, a companion cell important for fertilization that undergoes chromatin decompaction. Instead, other silencing mutants (*met1*, *ddm1*, *mom1*, *morc*) show loss of silencing in all pollen nucleus types and somatic cells. We show that TEs repressed by MBD5/6 gain chromatin accessibility in wild-type vegetative nuclei despite remaining silent, suggesting that loss of DNA compaction makes them sensitive to loss of MBD5/6. Consistently, crossing *mbd5/6* to *histone 1* mutants, which have decondensed chromatin in leaves, reveals derepression of MBD5/6-dependent TEs in leaves. MBD5/6 and SILENZIO thus act as a silencing system particularly important when chromatin compaction is compromised.

## In brief

Ichino et al. develop a method to profile gene expression of individual *Arabidopsis* pollen nuclei along their developmental trajectories. This leads them to discover that mutation of DNA methylation readers MBD5 and MBD6 causes loss of transposon silencing in pollen vegetative nuclei, likely because of their sensitized decompacted chromatin state.

## Graphical Abstract

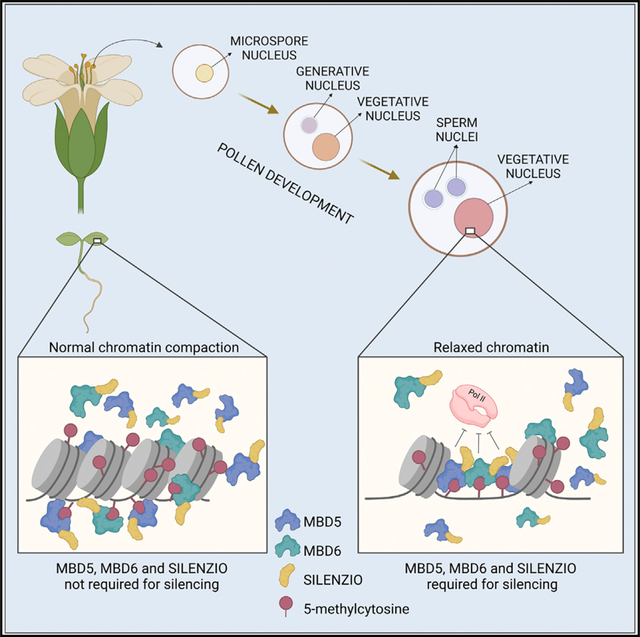

## INTRODUCTION

Eukaryotes have evolved numerous molecular strategies to establish and maintain silencing of genes and repetitive elements. Chemical modifications of histones and DNA work jointly with chromatin remodelers and histone variants to make DNA inaccessible to the transcriptional machinery.^1^ During development, some cells undergo a process of global epigenetic reprogramming, which entails the erasure of different layers of repression.^2^ An example of this occurs in the mammalian primordial germ cells, which undergo a genome-wide loss of DNA methylation to reestablish genomic imprints.^3^ This transient relaxation of repression creates a window of opportunity for mobile elements to reactivate and transpose, with potentially harmful consequences to genome integrity.^4^

In the flowering plant *Arabidopsis thaliana*, germline epigenetic reprogramming involves some changes in histone variants, histone tail marks, and non-CG methylation, but CG methylation levels are not globally erased like in mammals, thus allowing for transgenerational epigenetic inheritance of DNA methylation epialleles.^5,6^ On the other hand, extensive rewiring of chromatin structure and partial loss of CG methylation occur in reproductive “companion cells,” which function as supporting cells during fertilization and embryogenesis.^6^ The development of male gametophytes in *Arabidopsis* starts with a meiotic event that generates four identical haploid microspores from a Microspore Mother Cell. Each microspore subsequently undergoes an asymmetric mitotic division that produces a vegetative cell (VC) and a generative cell (GC). The GC is engulfed within the cytoplasm of the VC, thus creating the bicellular pollen grain. The GC then undergoes another round of mitosis that produces two identical sperm cells (SCs), forming the tricellular pollen grain. The SCs constitute the germline, while the VC is a supporting cell that is responsible for pollen tube growth, which allows delivery of the SCs to the female gametes for fertilization.^7^ The generation of pollen grains, from microspores to the tricellular stage, occurs over a time frame of about 3 days.^8–10^

The VC and the SC undergo remarkably different chromatin reorganization events that eventually lead to the formation of two small and highly compacted sperm nuclei (SN) and a very large and decondensed vegetative nucleus (VN). This striking difference is apparent in microscopy images of DAPI-stained mature pollen.^11^ More specifically, chromatin decompaction in the VN occurs, at least in part, because of the depletion of the linker histone H1, which begins at the late microspore stage^12^ and because of the active removal of the centromere-specific histone H3 variant (CenH3),^13^ which causes loss of centromere identity and dispersion of the H3K9me2 marked centromeric heterochromatin.^14^ Moreover, expression of the DNA glycosylase DEMETER (DME) in VN causes demethylation of some genes and transposable elements (TEs) in the CG sequence context.^15–17^ This active demethylation process is required to induce the expression of a small number of genes involved in pollen-tube function, which are critical for male fertility.^18,19^ On the other hand, the biological function of the VN chromatin decompaction is still debated, as it could occur to deliberately reactivate TEs to reinforce silencing in the germline,^15,20,21^ or to reactivate the thousands of copies of ribosomal RNA genes that are normally heterochromatinized and segregated in the chromocenters.^13^ Indeed, the VC is extremely metabolically active as it needs to rapidly elongate the pollen tube to find the ovule, which makes it one of the fastest growing eukaryotic cells known.^7^

We recently discovered that two *Arabidopsis* methyl reader proteins, Methyl-CpG-binding domains 5 and 6 (MBD5 and MBD6, or MBD5/6 for short), redundantly silence both genes with promoter methylation and TEs.^22^ MBD5/6 bind CG methylated DNA and repress transcription by recruiting the J-domain protein SILENZIO (SLN). While most genes and TEs repressed by DNA methylation are bound by MBD5/6, only a subset of them are derepressed in *mbd5/6* mutants.^22^ This led us to investigate what makes these loci sensitive to the loss of methyl readers and whether the derepression might be limited to certain cell types. Here, we show that loss of silencing in *mbd5/6* and in *sln* occurs specifically in VN of developing pollen grains. Given the special epigenetic state of this cell, we propose that the molecular function of MBD5/6 is revealed during pollen development because of diminished chromatin compaction, which makes the VN particularly prone to loss of silencing. Consistent with this, we found that the *mbd5/6* phenotype is enhanced in leaves by mutation of the linker histone H1, which causes chromatin decompaction in leaves.^23,24^ This result highlights the importance of evolving several redundant layers of silencing mechanisms to ensure the maintenance of TE repression. Furthermore, this work provides a comprehensive single-nucleus RNA-seq (snRNA-seq) dataset of *Arabidopsis* developing pollen nuclei, which has allowed insights into the transcriptome plasticity of male gametophytes.

## RESULTS

### The *mbd5/6* TE derepression phenotype is strongest in developing pollen

**Figure 1.**
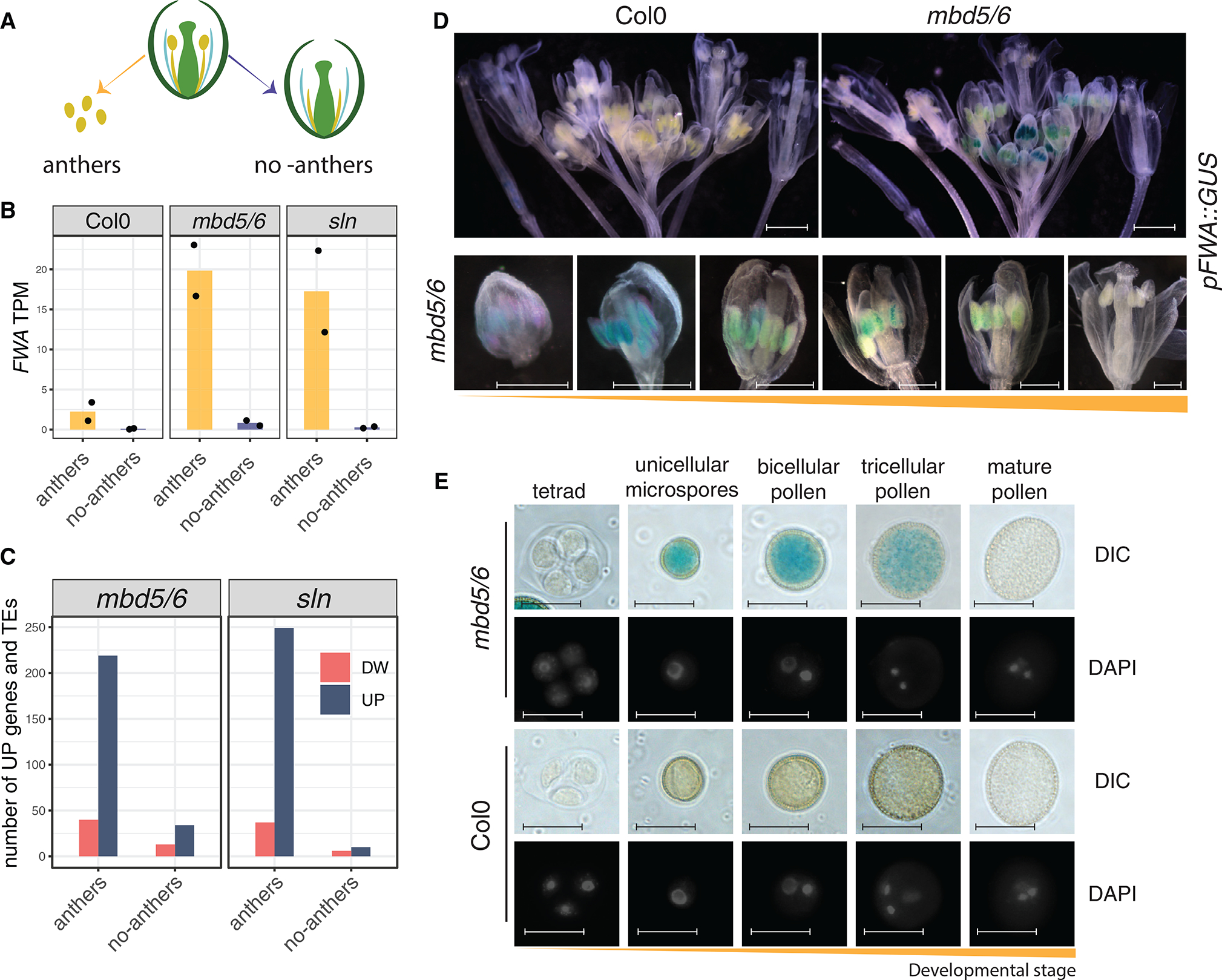
*FWA* derepression in *mbd5/6* is restricted to developing pollen grains (A) Cartoon depicting the dissection strategy: anthers were dissected from unopened flower buds and separated from the rest of the sample (“no-anthers”). (B and C) RNA-seq analysis of dissected fractions. (B) *FWA* expression (transcripts per million [TPM]), (C) number of up- or downregulated transcripts. (D) Microscopy images of GUS stained and cleared pFWA::GUS lines in the Col0 (wild type) or *mbd5/6* backgrounds (representative of more than 10 lines per genotype). Scale bars, 1,000 μm (upper panels) and 500 μm (lower panels). (E) Images of male gametophytes at the indicated developmental stages, isolated from *pFWA*::*GUS* lines in the Col0 (wild type) or *mbd5/6* backgrounds. Scale bars, 20 μm. See also Figure S1 and Table S1.

We previously observed that, in inflorescence tissue, the *mbd5/6* and *sln* mutant plants show a mild derepression of a small number of TEs and genes that are regulated by DNA methylation, among which a clear example was the gene *FWA*.^22^ *FWA* is normally methylated and silent in all tissues except for the endosperm, a tissue that surrounds and nourishes the embryo, where only the maternal copy is demethylated, thus allowing its monoallelic expression.^25^ A very small loss of methylation in the *FWA* promoter is also observed in the pollen VN.^17^ Consistently, recently published RNA-seq datasets detected low levels of *FWA* expression in microspores and bicellular pollen^26^ (Figure S1A). Given the specificity of the *FWA* expression pattern in reproductive tissues and given that our previously published *mbd5/6* and *sln* RNA sequencing (RNA-seq) experiments were performed with unopened flower buds, we wondered whether the loss of silencing occurred only in specific cell types within the flower. Therefore, we performed manual dissections of unopened flower buds to separate the anthers, which contain the developing pollen grains, from the rest of the flower bud, including carpels, petals, and sepals (named “no-anthers” for short) (Figures 1A and S1B). We then performed RNA-seq to compare the gene expression profiles in wild-type, *mbd5/6*, and *sln* (Table S1). We found that *FWA* derepression in *mbd5/6* and *sln* occurred exclusively in the anthers (Figure 1B). Furthermore, for both genotypes, differential gene expression analysis detected, in anthers, more than 200 upregulated and less than 40 downregulated transcripts (Figure 1C), which is consistent with the previously described function of this methyl reader complex in gene silencing.^22^ Notably, only 34 upregulated transcripts were detected in the no-anthers fraction of *mbd5/6* (10 in *sln*) (Figure 1C). Therefore, while we cannot rule out that MBD5/6 could regulate transcription in some rare cell types within the no-anthers fraction, the majority of the derepression signal measured in flower buds derives from the anther tissue.

To confirm this result with an orthogonal approach, we generated transcriptional reporter lines in which the *FWA* promoter drives the expression of the *beta-GLUCURONIDASE* (*GUS*) gene^27^ in the wild-type or *mbd5/6* genetic backgrounds. As previously shown, in wild-type plants we detected GUS staining only in young developing seeds, which contain the endosperm tissue, where *FWA* is endogenously expressed (Figure S1C). Instead, in *mbd5/6* mutant plants we detected a speckled pattern in anthers at different developmental stages (Figure 1D). The intensity of the GUS precipitate appeared strongest in early developmental stages and tended to fade away in the most mature anthers (Figure 1D). Further inspection confirmed that the GUS staining corresponded to the developing male gametophytes within the anther locules (Figure 1E). To understand which stages of pollen development display *FWA* expression, we performed DAPI staining of developing spores from the reporter lines. We found that GUS-positive pollen grains corresponded to all stages of development, from the uninuclear microspores to the bicellular and tricellular pollen grains, but the signal was no longer detectable in mature tricellular pollen (Figure 1E). Overall, these data indicate that, within the anther tissue, *FWA* is reactivated only in the male gametophytes, from early stages of pollen development, and its expression decreases in mature pollen. Given that pollen mitotic divisions occur over a timeframe of about 3 days,^8,9^ it is possible that *FWA* mRNA expression decreases before the loss of GUS protein, as we further address below.

*FWA* derepression only in the male gametophyte was unexpected because MBD5, MBD6, and SLN are all broadly expressed across many different tissues (Figure S1A). Therefore, their expression pattern cannot explain the pollen specificity of their phenotype. Moreover, in *Arabidopsis thaliana*, DNA methylation typically represses a largely overlapping set of genes and TEs in different tissues. The *FWA* gene, for instance, is very highly expressed both in vegetative and in reproductive tissues in *met1–3* mutant plants, in which CG methylation is lost genome-wide because of a mutation in the maintenance *DNA METHYLTRANSFERASE 1* (*MET1*) gene (Figure S2A).

**Figure 2.**
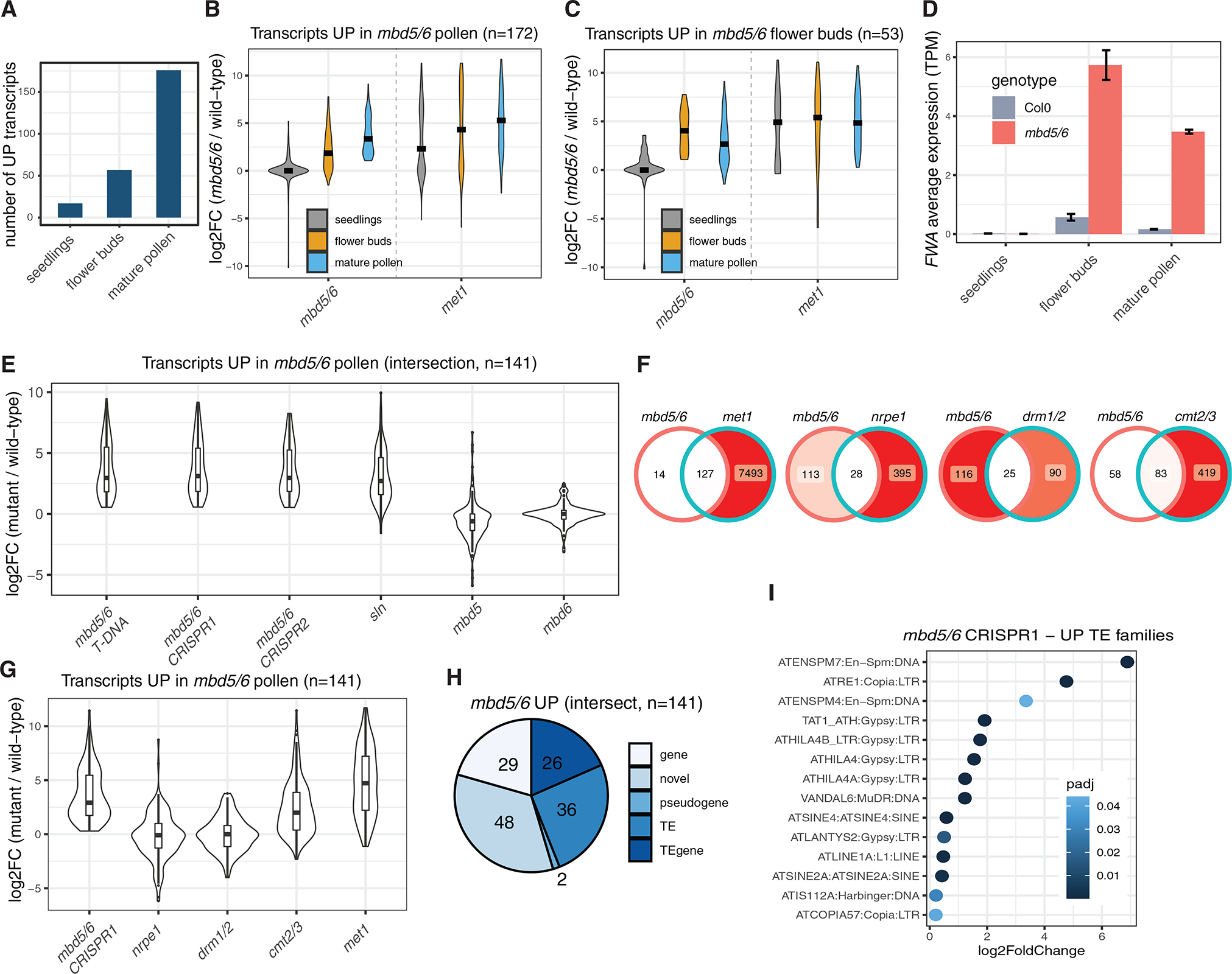
Tissue specificity of the *mbd5/6* transcriptional derepression phenotype (A) RNA-seq analysis of *mbd5/6* versus wild type. (B and C) Distribution of the RNA-seq log2 fold change (log2FC) for the transcripts (genes and TEs) that are upregulated in *mbd5/6* mature pollen (B) or unopened flower buds (C). Black line indicates the median. (D) Expression of *FWA* in Col0 and *mbd5/6* from the RNA-seq datasets in (A). (E and G) Comparison of the mature pollen RNA-seq pattern in different mutants. The overlayed violin plots and boxplots show the log2FCs of the upregulated transcripts that overlap between the three different *mbd5/6* mutants. (F) Overlaps between up-DEGs in mature pollen in the indicated mutants. (H) Features of the *mbd5/6* mature pollen upregulated transcripts. The “novel” transcripts were identified via a pollen transcript reannotation (see STAR Methods and Figure S3). (I) Analysis of significantly upregulated TE families in mbd5/6 (mature pollen). See also Figures S2–S4 and Tables S1 and S2.

The specificity of *FWA* derepression in *mbd5/6* for developing pollen grains prompted us to investigate and compare the gene expression patterns in seedlings, unopened flower buds, and mature pollen (see STAR Methods and Figures S2B and S2C for the mature pollen collection procedure). We detected 17 upregulated differentially expressed genes (up-DEGs) in *mbd5/6* seedlings, 57 in flower buds, and 176 in mature pollen (“DEGs” always indicates all transcripts [genes and TEs]) (Figures 2A and S2D). Only 3 out of 17 seedlings up-DEGs were upregulated in flower or pollen, while 33 out of 57 flower up-DEGs were upregulated in pollen, but only one of them in seedlings (Figures S2E and S2F). Similar results were obtained with *sln* (Figures S2D and S2G). When we plotted the distribution of the expression fold changes in the different tissues, we noticed that the pollen DEGs tend to have a positive fold change in flower buds as well, although lower in magnitude compared with pollen (Figures 2B and S2H). However, these loci are not upregulated in seedlings (Figures 2B and S2H). This is different from that observed in the methylation mutant *met1*, in which about half of the pollen DEGs are upregulated in seedlings as well (Figure 2B). Therefore, a portion of the *mbd5/6* pollen DEGs are repressed by DNA methylation in seedlings, but loss of MBD5/6 alone is not sufficient to reactivate them in that tissue.

We also noticed that a small number of genes, including *FWA*, are more strongly upregulated in *mbd5/6* flower buds than in mature pollen (Figures 2C, 2D, S2D, and S2I). This is consistent with the observations made with the *pFWA*::*GUS* reporter line, which showed loss of GUS signal in mature pollen (Figures 1C and 1D).

### The transcripts upregulated in *mbd5/6* pollen are genes and TEs repressed by CG methylation

Given that the mature pollen RNA-seq allowed detection of a higher number of DEGs compared with the previously published dataset,^22^ we investigated the features of these transcripts in greater depth. While inspecting the genome browser tracks, we noticed that several loci with aligned reads did not correspond to any annotated gene or TE (Figure S3). To detect these loci as DEGs, we performed a reannotation of transcripts based on our Col0, *mbd5/6*, *sln,* and *met1* mature pollen RNA-seq datasets (see STAR Methods and Figure S3). This allowed us to not only detect more DEGs but also refine the existing annotations, obtaining more accurate transcriptional start, end, and splicing sites (Figure S3). We then used these annotations to analyze a mature pollen RNA-seq dataset that includes the *mbd5* and *mbd6* single mutants, three different *mbd5/6* double mutants, *sln*, *met1*, the RdDM mutants *drm1 drm2* (*drm1/2*), and *nrpe1*, which lose non-CG methylation at RdDM sites, and the *cmt2 cmt3* (*cmt2/3*) mutant, which affects methylation at heterochromatic TEs.^28^

We obtained about 200 up-DEGs in the *mbd5/6* samples and in *sln*, while less than 20 in the *mbd5* and *mbd6* single mutants (Figures 2E, S4A, and S4B; Table S2). This is consistent with our previous observation that MBD5 and MBD6 are genetically redundant, and that SLN is the repressor acting downstream of the methyl readers.^22^ The *mbd5/6* up-DEGs (n = 141, intersection between the three *mbd5/6* mutants) were mostly a small subset of the *met1* up-DEGs (127/141) and overlapped with the *cmt2/3* up-DEGs (83/141), but not as much with the DEGs of the RdDM mutants *nrpe1* and *drm1/2* (28/141, 25/141) (Figure 2F). Consistently, these genes had an overall positive fold change in *met1* and *cmt2/3*, but not in *nrpe1* and *drm1/2* (Figure 2G). This indicates that the MBD5/6 targets are mostly heterochromatic loci, as previously observed in flower buds^22^. While some of them, such as *FWA*, are euchromatic and methylated by the RdDM machinery, they are typically not upregulated in RdDM mutants, but only in *met1*, suggesting that they are mainly repressed by CG methylation.

The *met1* up-DEGs included both loci that are promoter methylated and unmethylated, the latter being likely indirect targets (FigureS4C). In contrast, almost all the *mbd5/6* up-DEGs were promoter methylated and were not expressed in wild-type flowers (Figures S4C and S4D). Consistently, we found that most of them were TEs, TE genes (transposon related genes as defined in the TAIR10 genomic annotations), or novel transcripts obtained with our pollen reannotation pipeline (Figure 2H). TE family analysis showed that the MBD5/6 targets include both retrotransposons and DNA transposons, both of which are upregulated in *met1* as well (Figures 2I, S4E, and S4F).^29^ Therefore, MBD5 and MBD6 do not regulate a specific class of TEs, but broadly regulate TE families that are repressed by CG methylation.

We also identified a few functional genes that, similarly to *FWA*, have promoter methylation and are repressed by MBD5/6 and MET1 (Figure S4G). One of them was ANTAGONIST OF LIKE HETEROCHROMATIN PROTEIN 2 (ALP2), a gene domesticated from a transposon, which antagonizes the function of POLYCOMB REPRESSIVE COMPLEX 2 (PRC2).^30^

Overall, these datasets show that, among the investigated tissues, the transcriptional phenotype of *mbd5/6* and *sln* is strongest in pollen, and that the MBD5/6 targets are genes and TEs repressed by CG methylation. Given that pollen grains contain different nucleus types, we next used snRNA-seq to pinpoint in which specific nuclei the transcriptional changes occurred during pollen development.

### snRNA-seq reveals the transcriptional landscape of developing male gametophyte nuclei

**Figure 3.**
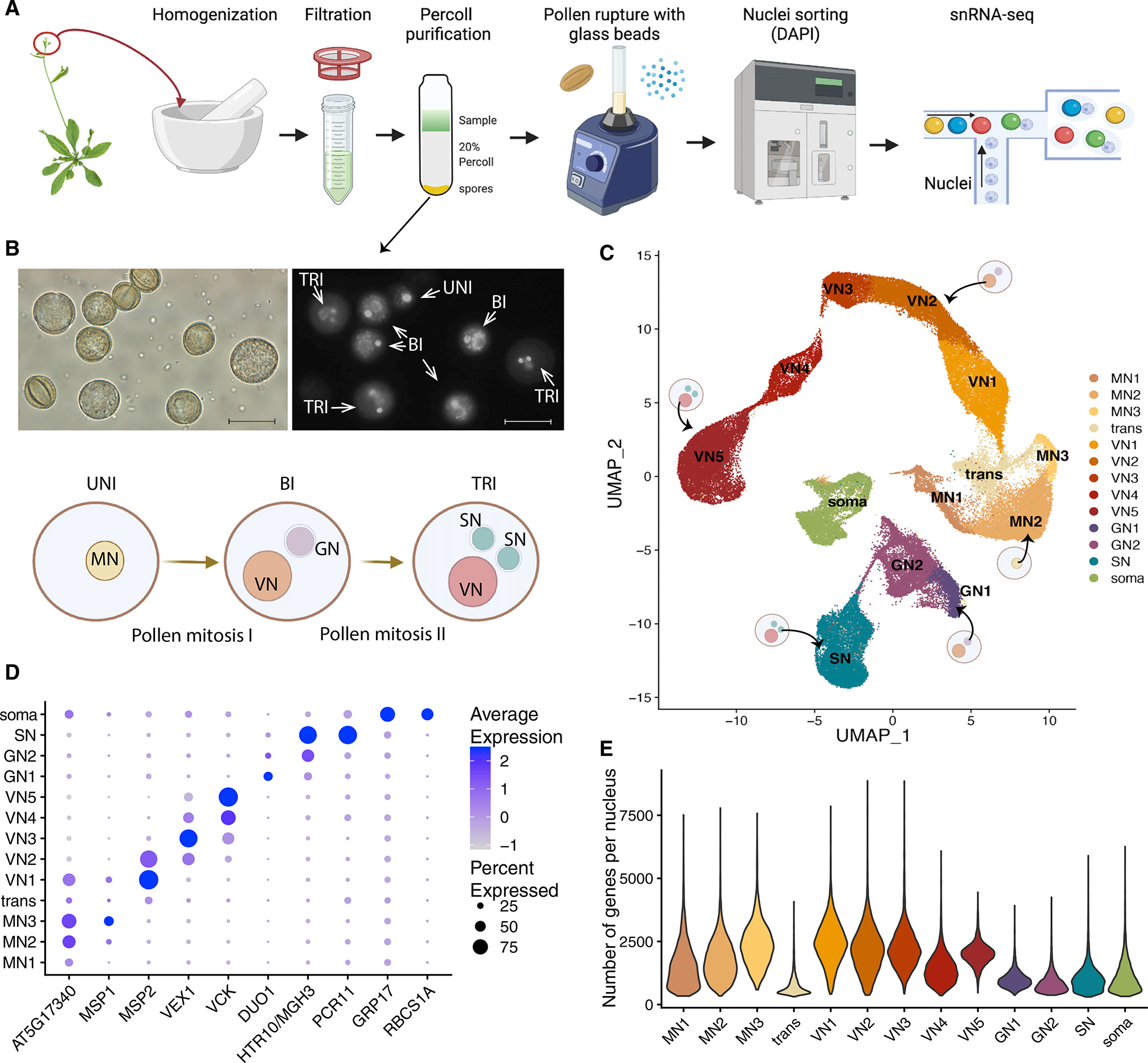
Single-nucleus RNA-seq of developing male gametophytes (A) Cartoon representation of the protocol developed for this study. (B) Upper panels: representative image of the mixed spores sample obtained after gradient centrifugation. Left: DIC. Right: DAPI. Scale bar, 20 μm. Lower panel: cartoon representation of pollen development. “Uni,” “bi,” and “tri” indicate uninuclear, bicellular, and tricellular pollen stages. (C) UMAP of all integrated snRNA-seq datasets with clusters annotations. (D) Dot plot showing the cluster specificity of the expression of known markers. Dot size: percentage of cells in which the gene was detected. Dot color: scaledaverage expression. (E) Violin plots showing the distribution of the number of unique genes detected per nucleus, in each cluster. See also Figure S5 and Tables S3–S6.

We developed a method to capture the transcriptome of each nucleus of developing pollen grains (Figure 3A). In brief, we isolated male gametophytes from *Arabidopsis* inflorescences by gentle homogenization of the tissue, and we purified the mixed spores from contaminating tissue by centrifugation over a Percoll cushion. Visual inspection of the sample confirmed that this method allowed isolation of mixed-stage pollen grains, from the uninuclear microspores to the tricellular mature pollen (Figure 3B). To break the pollen wall and release the nuclei, we vortexed the sample intermittently in the presence of glass beads, as previously done for VN and SN isolation.^11,31^ We then sorted the nuclei based on DAPI to purify them from the debris (Figures S5A–S5C), and we performed single-nucleus RNA-seq with the 10X Genomics Chromium platform. The preprocessing and quality control of the datasets were done with Seurat using standard workflows (STAR Methods section “analysis of snRNA-seq,” Table S3).

To visualize the data, we performed dimensionality reduction via Uniform Manifold Approximation and Projection (UMAP) followed by unsupervised clustering (Figure 3C). Inspection of several known markers of the different pollen nucleus types allowed the identification of clusters corresponding to microspore nuclei (MN), VN, generative nuclei (GN), SN, and some contaminating nuclei (labeled “Soma”), likely deriving from somatic tissues that surround the developing pollen grains in the anthers (Figures 3C and 3D). We identified marker genes for each cluster (Table S4). The cluster identity assignments were also confirmed by selecting the top 20 markers for each cluster, and plotting their normalized expression in several RNA-seq datasets from the EVOREPRO database which were generated from bulk RNA-seq of whole pollen grains at different developmental stages^26^ (Figure S5D). As expected, the expression profile of whole pollen correlates with that of the VN, and not with SN or GN, because most of the pollen mRNA content derives from the cytoplasm of the VC.^19^

To put the expression defects of *mbd5/6* and *sln* in the context of development, we first characterized the wild-type expression patterns of all nucleus types in this dataset. The next section describes the nuclei clusters, before delving into the transcriptional changes found in *mbd5/6* and *sln*.

We observed that the VN were distributed in the UMAP along a clear developmental trajectory, proceeding from the bicellular stage to the tricellular and mature pollen (labeled as VN1 to VN5) (Figure 3C). This was indicated by the initial expression of *MICROSPORE-SPECIFIC PROMOTER 2* (*MSP2*), a VN marker of the early bicellular stage,^32^ followed by the tricellular stage VN marker *VEGETATIVE CELL EXPRESSED 1* (*VEX1*),^33^ and then by the expression of *VEGETATIVE CELL KINASE 1* (*VCK1*), which is strongest in the latest stages of mature pollen^34^ (Figure 3D). Based on this observation, we generated a developmental trajectory using Monocle3^35^ to rank VN according to their predicted pseudotime, which represents the amount of progress that each nucleus has made toward differentiation (Figures S5E and S5F). We then identified the genes that change as a function of pseudotime (see STAR Methods), and performed hierarchical clustering to find patterns, which revealed different waves of transcriptional regulation (Figure S5G; Table S5). After performing gene ontology (GO) analysis, we found that the genes expressed in early stages and then gradually downregulated were enriched in several GO terms related to mRNA processing and ribosome biogenesis, the genes that were transiently upregulated in the middle of the trajectory were related to Golgi vesicle transport and endocytosis, and the genes upregulated in the later stages were associated with cell tip growth and pollen tube development GO terms (Figures S5G; Table S5). This analysis suggests that VN initially work toward building the machinery required for extensive transcription and translation, and later use it to produce the proteins needed for membrane expansion and pollen tube growth, an extremely demanding metabolic process entailing extensive membrane trafficking.^7^ The high transcriptional activity of these nuclei is revealed also by the higher average number of genes detected compared with SN and GN (Figure 3E).

We then identified the GN nuclei through the master transcription factor *DUO POLLEN 1* (*DUO1*), which is expressed in the GN right after pollen mitosis I^36,37^ (Figure 3D). The SN nuclei instead were marked by sperm cell specification genes that are activated by DUO1, such as *MGH3/HTR10* and *PCR11*.^37^ While *PCR11* is known to be sperm cell specific, *MGH3/HTR10* was shown to be expressed in the GN of the late bicellular pollen as well as in SN,^38^ and indeed we were able to detect strong expression of this gene in GN2 (Figure 3D). Several other known DUO1 targets were also detected in GN2 and SN clusters (Figure S5H).^37^ The relative expression patterns of these genes suggest that the GN2 cluster comes after GN1 in the developmental timeline (Figures S5H and S5I). GO analysis of the GN cluster markers highlighted terms related to cell cycle and chromosome organization (Table S6).

We identified three clusters corresponding to MN (MN1, MN2, and MN3). These clusters express the previously described microspore specific marker AT5G17340,^39^ while *MICROSPORE SPECIFIC PROTEIN 1* (*MSP1*)^32^ was only detectable in MN2 and MN3 (Figure 3D). The expression pattern of these two genes suggests that these nuclei are distributed along a developmental trajectory proceeding from MN1 to MN3. Consistently, an analysis of known cell-cycle markers^40^ revealed that MN1 is enriched in S-phase markers, while MN3 is enriched in M-phase markers, which supports the directionality of the trajectory (Figure S5J). Indeed, *MET1*, which is highly expressed in S-phase to methylate the newly synthesized DNA,^41^ was also highly expressed in MN1 (Figure S5K). GO analysis revealed that the MN3 cluster, in addition to cell-cycle terms, was also enriched in terms related to ribosome biogenesis, suggesting that late microspores are already beginning to establish a transcriptional program aimed at increasing the metabolic output of the upcoming VN (Table S6).

The UMAP analysis identified a group of cells localizing between MN and VN1 clusters that, despite having a gene expression profile similar to MN, were characterized by very low numbers of genes detected per nucleus and few cluster markers (Figures 3C and 3E; Table S3 and S4). We named this cluster “transitory” (“trans”) because we envision that it could correspond to cells that are in the process of undergoing mitotic division, and therefore might be going through a transient global decrease in transcriptional output.^42^ However, we acknowledge that these cells could also correspond to damaged nuclei or chromatin fragments, and therefore we did not include this cluster in our downstream analyses.

We noticed that one of the top markers for the MN clusters was *DEMETER-LIKE PROTEIN 3* (*DML3*), a poorly characterized DNA demethylase enzyme (Table S4; Figure S5K). This prompted us to investigate the family of *Arabidopsis* demethylases. As expected, we observed a strong enrichment of *DEMETER* (*DME*) in early VN (Figure S5K), consistent with DME’s known role in demethylating some genes that are required for pollen tube growth.^16,18^ ROS1 (DML1) has also been shown to participate in the process of demethylation in VN. Consistently, we observed that its expression was strongest in late MN and VN1. *DML3* instead was strongly expressed in MN1 and MN2. Interestingly *DML2* was mostly enriched in GN1 (Figure S5K), suggesting that different demethylases might have cell-type-specific functions during pollen development.

We next inspected the expression patterns of DNA methyltransferases. We observed that *CMT1* was only expressed in GN1, while its homolog *CMT3,* which has CHG methyltransferase activity, was expressed in MN, SN, and GN (Figure S5K). *CMT2* instead, which has CHH specificity, was most highly expressed in early VN, and therefore it is likely responsible for the known increase in CHH methylation levels in VN, given that the other CHH methyltransferases, *DRM1* and *DRM2*, were very lowly expressed (Figure S5K).^15,43^ Lastly, *MET1*, as previously mentioned, has an expression pattern that strongly correlates with cell cycle, being most highly expressed in MN1, GN, and SN.

Inspection of the machinery required for the deposition and removal of histone marks revealed that some enzymes were broadly expressed while others were restricted to specific lineages (Figure S5K). Of note, the H3K27 methyltransferases *CURLY LEAF* (*CLF*) and *SWINGER* (*SWN*) were enriched in VN and depleted from SN, which is consistent with a recent report of the erasure of H3K27 in SN.^44^

We also looked at the expression patterns of the small RNA machinery, given its prominent role in pollen biology.^45,46^ As previously described,^45,46^ we found that *AGO5* was expressed in GN and *AGO1* in microspores and VN (Figure S5K).

The role of histone variants in shaping the epigenome of male gametophytes has gained increasing attention in recent years.^47^ In addition to *MGH3/HTR10*, a well-known sperm-specific H3 variant (H3.10), we validated the sperm specificity of *H2B*.8 (*HTB8*), which was recently found to mediate chromatin condensation in sperm to reduce nuclear size^48,49^ (Figure S5L). Interestingly, *H2B*.*5* and *H2B.7* were enriched in sperm as well, and these variants have not been functionally analyzed yet (Figure S5L). H2B.10 was instead preferentially expressed in the late VN, as reported previously.^48^

Overall, our snRNA-seq dataset provides a comprehensive overview of gene expression profiles throughout the development of *Arabidopsis* male gametophytes and constitutes a source for future studies.

### The MBD5/6 targets are derepressed in the VN lineage

**Figure 4.**
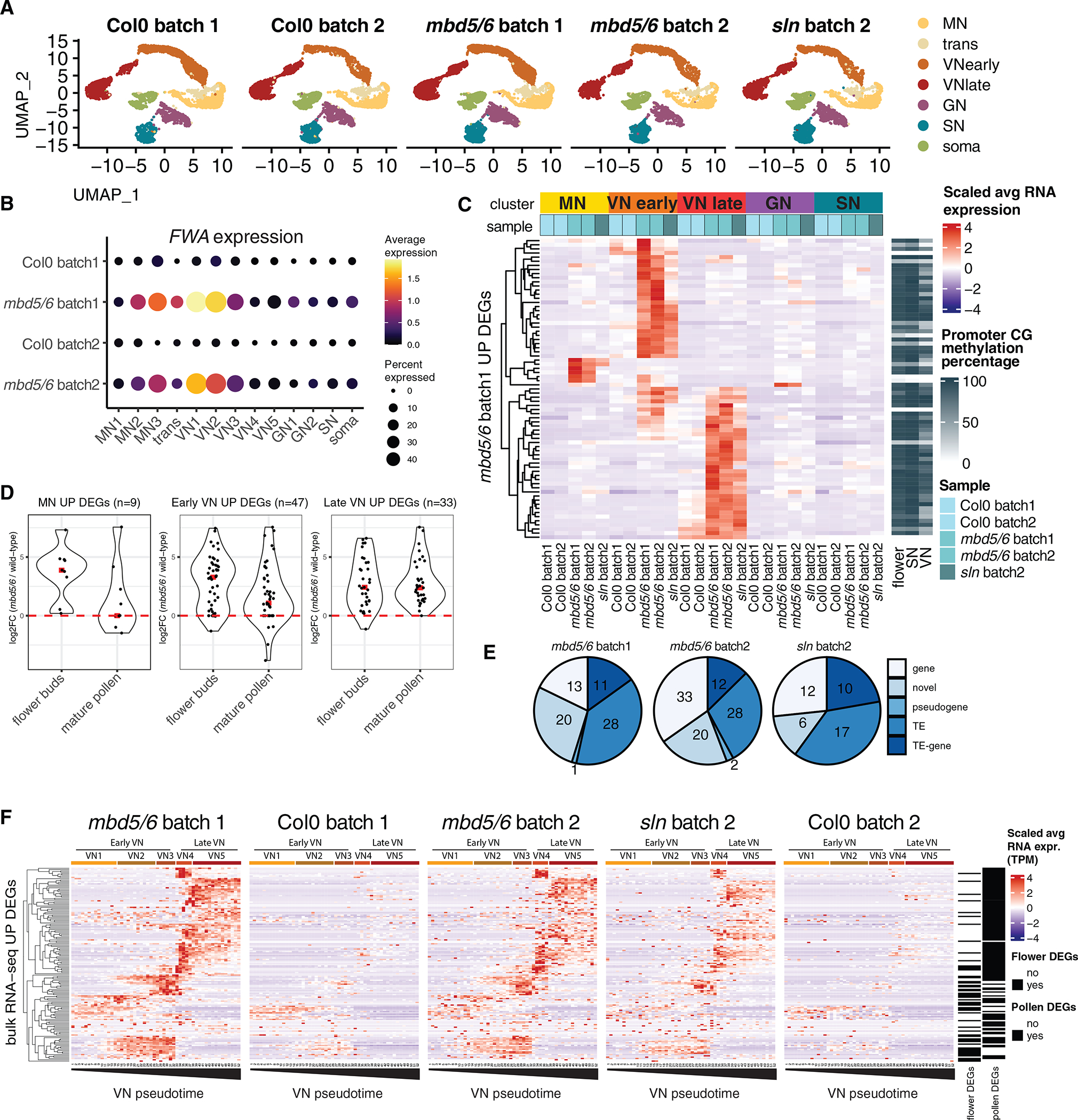
Transcriptional derepression in *mbd5/6* is limited to the MN/VN lineage (A) UMAP plots of the integrated Col0, *mbd5/6*, and *sln* snRNA-seq datasets. “Batch 1” and “Batch 2” indicate two independent experiments. (B) Dot plot of *FWA* expression in Col0 and *mbd5/6*, at each cluster. Dot size: percentage of cells in which *FWA* was detected. Dot color: scaled average expression. (C) Heatmap of the *mbd5/6* batch 1 upregulated transcripts (union of all cluster). Shown is the snRNA-seq scaled average expression level per cluster in the indicated samples. Right columns: wild-type CG methylation percentage at promoters. (D) Violin plots of expression changes in *mbd5/6* obtained by bulk RNA-seq in flowers or mature pollen, for the indicated groups of snRNA-seq genes (red dash: median). “DEGs” indicates all transcripts (genes and TEs). (E) Classification of the upregulated transcripts for each experiment (union of all clusters). (F) Heatmap of scaled snRNA-seq expression along the VN pseudotime trajectory (see Figure S5E). Shown is the union of flower bud and mature pollen *mbd5/6* upregulated transcripts obtained by bulk RNA-seq (see Figure S2). See also Figure S6 and Table S7.

To investigate the cell-type specificity of *mbd5/6* derepression during pollen development, we performed snRNA-seq of Col0, *mbd5/6*, and *sln* mutant plants (Figure 4A). We first looked at the *FWA* gene: we found that its expression in *mbd5/6* begins increasing in the late microspore stage, peaks in the VN of bicellular pollen, and then starts decreasing in the late bicellular stage of VN (Figure 4B), consistent with the results obtained with the *pFWA*::*GUS* reporter line. This suggests that GUS signal in early tricellular pollen may be in part due to persistent protein expression when the mRNA levels are decreased (Figures 1C and 1D). *FWA* expression was very mild in *mbd5/6* GN and SN, suggesting that its upregulation is mostly restricted to the MN/VN lineage.

To test whether other MBD5/6 targets showed the same pattern as *FWA*, we performed a differential gene expression analysis comparing *mbd5/6* with wild type for each cluster, including samples from two independent experiments. We employed a stringent approach for calling DEGs to limit the false positives (see STAR Methods and Figure S6). We observed that the clusters corresponding to the same nucleus type (such as VN4 and VN5) had a similar pattern of differential expression (Figure S6F), therefore we decided to group together these clusters for ease of visualization, and only display MN, early VN, late VN, GN, and SN groups in a scaled heatmap (Figures 4A and 4C). We observed that few of the *mbd5/6* and *sln* DEGs were upregulated in microspores, while most of them were upregulated either in the early or in the late VN clusters, and they were not strongly upregulated in GN or SN (Figures 4C and S6). To validate this observation, we analyzed the expression of these DEGs in the bulk RNA-seq datasets performed on unopened flower bud tissue and on mature pollen. We reasoned that the microspore and early VN DEGs should be more strongly upregulated in the “unopened flower bud” bulk RNA-seq sample, which contains anthers with developing pollen grains, compared with the mature pollen sample. Indeed, the result confirmed the prediction, validating the stage specificity of the DEGs (Figure 4D). As expected, these loci were characterized by promoter methylation and included several TEs and novel annotations (Figures 4C and 4E). The number of DEGs obtained from the snRNA-seq data was lower than that obtained from the bulk RNA-seq, likely because of the high variability in single-cell data (see STAR Methods and Figure S6). However, our approach was able to identify high confidence and reproducible DEGs, including *FWA* and several TEs.

We next investigated the dynamics of expression of the DEGs along the VN developmental trajectory. We plotted the expression levels of the loci that were defined as upregulated in *mbd5/6* by mature pollen or flower buds bulk RNA-seq (n = 171) (Figure 4F). Although most of them were not called as snRNA-seq DEGs based on our stringent approach (129/171, Figure S6G), visual inspection revealed that 137/171 were upregulated in *mbd5/6* or *sln* compared with the controls (Figure 4F). We observed a progressive upregulation along the trajectory, with 54 genes being expressed in early VN and then silenced, and 83 genes being upregulated in late VN (Figure 4F). Interestingly, this same pattern, but with much lower magnitude, was observed in wild-type as well, whereby genes upregulated early in *mbd5/6* or *sln* also tended to be detected in the early stages in wild-type, and the late upregulated genes in the later stages. This suggests that the stage specificity of the derepression likely reflects the expression of needed developmental stage specific transcription factors.

Overall, the snRNA-seq data revealed that loss of silencing in *mbd5/6* and *sln* begins in the late microspore stage and becomes progressively more prominent in the VN along its developmental trajectory, while GN and SN are not affected.

### Other silencing mutants show broad derepression across all pollen nucleus types

**Figure 5.**
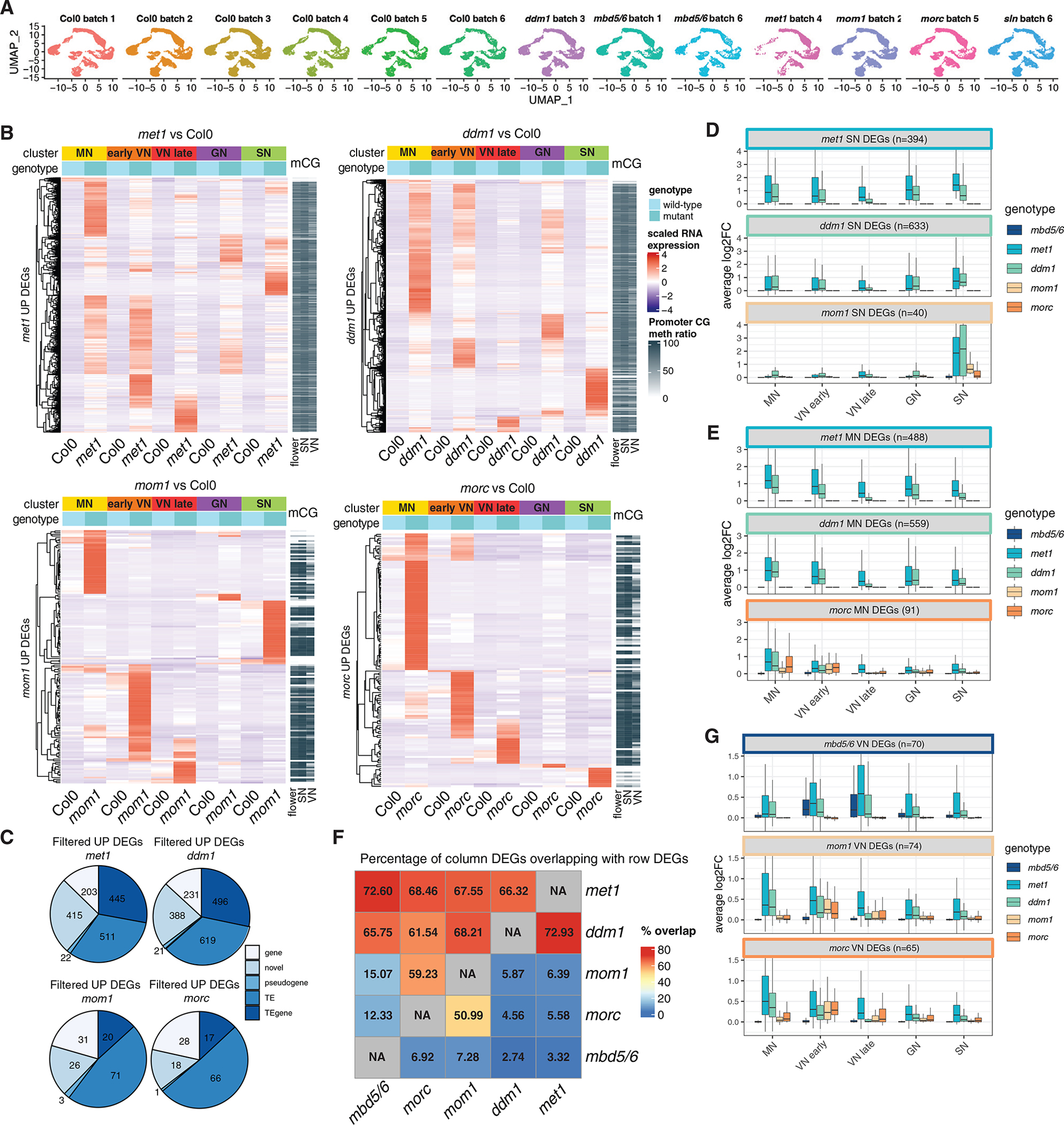
*met1*, *ddm1*, *mom1*, and *morc* mutants display loss of silencing in all pollen nucleus types (A) UMAP plots of all the snRNA-seq datasets used in this study. (B) Heatmaps of the genes and TEs upregulated in the indicated mutants (union of all clusters). Shown is the snRNA-seq scaled expression level of the cluster averages in the indicated samples. Right columns: wild-type CG methylation percentage at promoters. (C) Classification of the upregulated transcripts for each mutant (union of all clusters). (D, E, and G) Boxplots of snRNA-seq expression changes. (F) Heatmap representation of the percentage of DEGs upregulated in the mutant indicated in the column label (“column DEGs”) that overlap with DEGs upregulated in the mutant annotated in the row label (“row DEGs”). For instance, 72.6% of the *mbd5/6* upregulated DEGs overlap with the *met1* upregulated DEGs. “DEGs” always indicates all transcripts (genes and TEs). See also Table S7.

Intrigued by the VN specificity of the MBD5/6 targets, we wondered whether mutations in other genes involved in silencing of methylated DNA might show a similar or different pattern. To address this, we performed male gametophyte snRNA-seq with a panel of other well-characterized mutants that have TE derepression. We selected two “strong” mutants characterized by an extensive loss of methylation and high numbers of derepressed TEs, *met1* and *ddm1*, and two “weak” mutants that have fewer derepressed TEs, and very limited loss of methylation, *mom1* and *morc1*,*2,4,5,6,7 hextuple* (hereafter named “*morc*”). As previously noted, *met1* displays a genome-wide loss of CG methylation and limited loss of non-CG methylation, with strong TE derepression, while mutations in the nucleosome remodeler *DECREASE IN DNA METHYLATION 1* (*DDM1*) cause loss of CG and non-CG methylation only in pericentromeric heterochromatin, and strong derepression of heterochromatic TEs.^50,51^ Mutations in *MORPHEUS*′ *MOLECULE1* (*MOM1*) instead cause a mild derepression of heterochromatic TEs with very limited loss of DNA methylation, but the exact mechanism of MOM1-mediated silencing is not understood.^52,53^ Similarly, mutating the entire family of MORC GHKL ATPases causes derepression of some heterochromatic TEs that do not display a strong loss of methylation.^54^ We investigated each mutant with a matched wild-type control (Figure 5A). In the *met1* sample we obtained relatively fewer cells corresponding to the most mature stages of pollen development (VN4, VN5, SN) probably due to the phenotypic defects in flower development in the *met1* plants (Figure 5A and Table S3).

In all cases, we detected DEGs distributed among all clusters (Figures 5B and S7). For all mutants, about 75% of the DEGs were TEs or novel transcripts, characterized by promoter methylation (Figures 5B and 5C). The heatmap of the expression of these DEG among the different cluster groups highlighted how, unlike *mbd5/6* and *sln*, these mutants showed transcripts strongly upregulated in every cell type in developing and mature pollen (Figures 5B and S7). The *morc* mutant was particularly enriched in microspore-specific DEGs, suggesting that MORC proteins could play an important role in the early stages of pollen development. The *mom1* mutant instead was relatively more enriched in SN specific DEGs compared with the other mutants. We noticed that, while the *ddm1* and *met1* SN DEGs were similarly upregulated in all clusters, the *mom1* SN DEGs were instead more strongly upregulated in SN compared with the other clusters, even in *ddm1* and *met1* (Figure 5D). This suggests that, in weaker mutants, such as *mom1*, only the loci that have strong, cluster specific promoters tend to be derepressed. Consistently, the *morc* MN DEGs also had a clear cluster specificity, because even in *ddm1* and *met1 t*hey were more strongly upregulated in MN compared with the other clusters (Figure 5E).

We next compared the lists of upregulated DEGs of the different mutants, and we observed that, while a large proportion of the MBD5/6 targets were upregulated in *met1* and *ddm1*, they were not upregulated in *mom1* or in *morc* (Figure 5F). Similarly, the MOM1 and MORC targets were mostly a subset of the MET1 and DDM1 targets, and they largely overlapped with each other, but were not upregulated in *mbd5/6* (Figure 5F). Consistently, when plotting the distribution of the fold changes we found that the *mbd5/6* VN DEGs were not upregulated in *mom1* or in *morc*, while the *mom1* and *morc* VN DEGs were not upregulated in *mbd5/6* (Figure 5G). Therefore, the MBD5/6 targets constitute a unique subset of the loci regulated by DNA methylation. This prompted us to further investigate the features of these loci with the goal of understanding why this is the only mutant displaying a strong VN specificity.

### Loss of histone H1 uncovers a derepression phenotype of *mbd5/6* and *sln* in non-reproductive tissues

**Figure 6.**
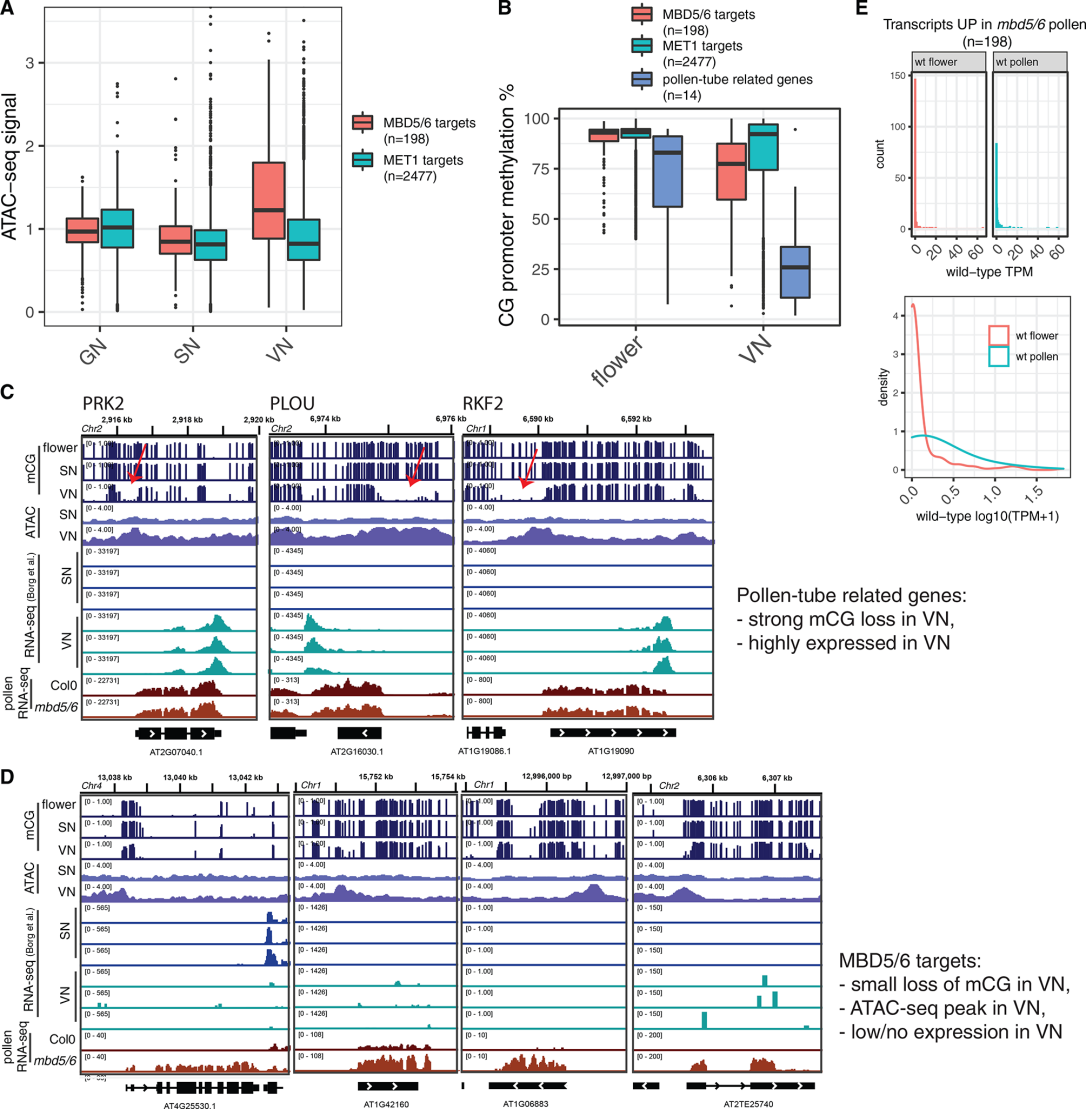
The MBD5/6 targets are characterized by increased accessibility in the wild-type VN (A) Boxplot showing average ATAC-seq signal around the transcriptional start site of the MBD5/6 targets (loci with promoter methylation upregulated in *mbd5/6* mature pollen) or MET1 targets (loci with promoter methylation, upregulated in *met1* but not in *mbd5/6* mature pollen). The VN, GN, and SN ATAC-seq data are publicly available.^19^ (B) Boxplots of average CG promoter methylation at the loci defined in (A) and at 14 manually curated genes that are demethylated by DME in the VN (list in STAR Methods).^18,19^ (C and D) Genome browser tracks showing examples of genes required for pollen fertility (C),^18^ and genes/TEs repressed by MBD5/6 (D). Red arrows: strong loss of CG methylation that allows expression in the wild-type VN. (E) Histograms of the wild-type expression level (TPM) in unopened flower buds and mature pollen, for the loci derepressed in *mbd5/6*. The lower plot shows the smoothened trend of the data to highlight a mild increase in expression in pollen.

The VN is a large nucleus and adopts a very peculiar chromatin state, characterized by global decondensation and loss of heterochromatic chromocenters.^12,13^ We wondered whether this unusual relaxed chromatin state could be related to the VN specificity of the loss of silencing in *mbd5/6*, as derepression coincides temporally with the depletion of H1 from VN chromatin.^12^ Given that only a small subset of the transcripts that are derepressed in *met1* are reactivated in *mbd5/6* as well, we tested whether these loci are characterized by higher levels of chromatin accessibility. We utilized a recently published dataset including assay for transposase-accessible chromatin sequencing (ATAC-seq) performed on nuclei isolated from wild-type GN, SN, and mature VN.^19^ We observed that, in the wild-type VN, there was a clear increase in chromatin accessibility at the MBD5/6 targets but not at the MET1-specific targets (which are not upregulated in *mbd5/6*) (Figure 6A). This supports the hypothesis that the MBD5/6 targets might be particularly prone to loss of silencing in VN because they gain accessibility in this cell type. The MBD5/6 targets also tended to lose more CG methylation in VN compared with the MET1 targets, possibly because the openness of their promoters facilitates access of DME, as suggested previously^12^ (Figure 6B). However, while the DME-mediated demethylation of specific pollen fertility genes in the VN is very extensive, causing these genes to be highly expressed and not regulated by MBD5/6, the loss of methylation at the MBD5/6 targets was milder, and only led to very low levels of expression in wild-type VN (Figures 6B–6E).

**Figure 7.**
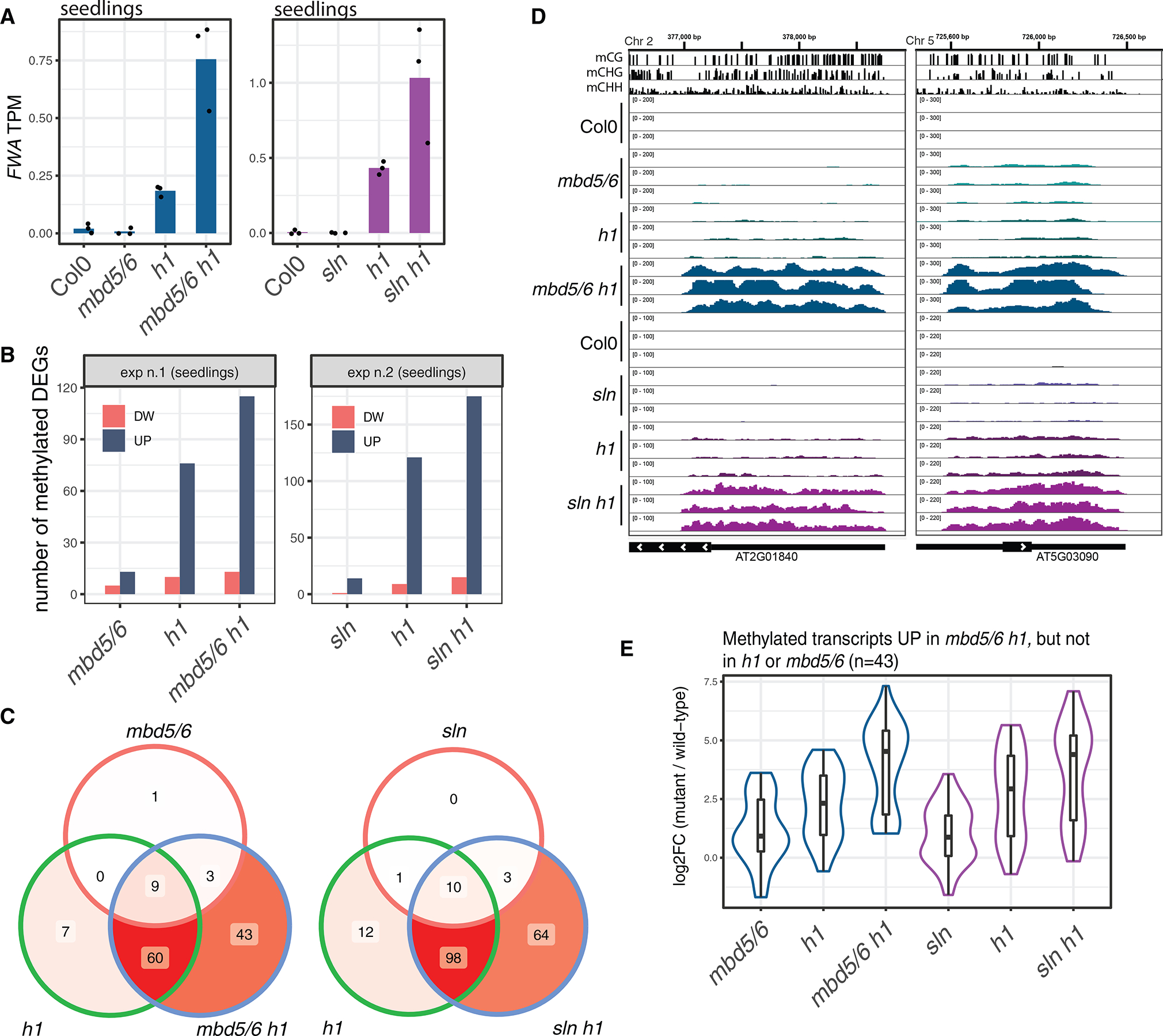
H1 mutation enhances the *mbd5/6* derepression phenotype in seedlings (A) Bar plots of average *FWA* expression in the indicated genotypes (seedlings RNA-seq). Dots: individual replicates. (B) Number of up- or downregulated transcripts for each mutant. Only loci with average CG promoter methylation higher than 40% are shown. (C) Venn diagrams of upregulated methylated transcripts. (D) Examples of two loci that are enhanced by combined loss of MBD5/6 and H1 (BS-seq: flower buds, RNA-seq: seedlings). (E) Overlay of violin plots and boxplots showing expression changes for the indicated transcripts.

This analysis suggested that the reason why there is no detectable TE derepression in *mbd5/6* and *sln* seedlings could be that chromatin compaction compensates for the loss of the methyl readers and is sufficient to maintain gene silencing. To test this idea, we crossed *mbd5/6* and *sln* with the *h1*.*1/h1.2* double mutant (here by named *h1* for short), which has been shown to have decondensed chromatin in seedlings.^12,23,24^ After performing RNA-seq in seedlings we found that the *FWA* gene, which is not expressed in *mbd5/6* or *sln* seedlings, was very mildly expressed in *h1*, and clearly enhanced in *mbd5/6 h1* and *sln h1* (Figure 7A). At a global level, we also observed an enhancement of derepression of methylated loci in the higher-order mutants compared with *h1*, *mbd5/6*, or *sln* alone, indicating that, when chromatin compaction is impaired as in *h1*, the function of MBD5/6 and SLN is revealed in seedlings (Figures 7B–7D). We found about 50 genes that were significantly upregulated only in *mbd5/6 h1* or *sln h1* (Figure 7C). Interestingly, these sites also show positive, though milder, upregulation in *mbd5/6* and *sln* alone (Figure 7E). Therefore, they tend to be mildly upregulated in *mbd5/6*, even in the presence of H1, but their expression is increased when H1 is depleted. These results suggest that MBD5/6 and SLN have gene-silencing functions in a broader range of tissues, but that redundancy with other silencing pathways prevents upregulation of target genes in most cells other than the VN.

## DISCUSSION

The complexity of gene-silencing mechanisms reflects a strong evolutionary pressure to preserve genome integrity by repressing mobile elements in eukaryotic organisms. The redundancies between different silencing pathways make these mechanisms challenging to study; indeed, a role for *Arabidopsis* methyl reader proteins in gene silencing downstream of DNA methylation has only been discovered recently.^22^ In this study we show that the function of MBD5, MBD6, and SILENZIO becomes evident in the VN of pollen, a specific cell type that has diminished function of important silencing factors that normally maintain chromatin compaction. To facilitate an extremely high transcriptional and metabolic activity, the vegetative cell undergoes dramatic chromatin decondensation, which makes it vulnerable to increased transposon activity. Our results suggest that the MBD5/6 methyl readers are important to combat the upregulation of transposons that become accessible to the transcriptional machinery in this fragile, but reproductively crucial, cell type.

In addition to TEs, a limited number of functional genes were found to be upregulated in *mbd5/6*, and these genes are not known to be involved in pollen development (Figure S4G). Indeed, we did not observe any pollen developmental defect in *mbd5/6*. Strong TE derepression instead could interfere with the biological activities of the VN by, for instance, sequestering energy from other important cellular processes required for pollen tube development. Future studies aimed at investigating pollen function and fertilization could reveal functional defects in *mbd5/6* pollen. The *met1* mutant instead had a stronger transcriptional deregulation phenotype and displayed a decrease in the relative number of mature pollen grains, possibly due to strong transposon derepression or deregulation of specific genes. It is likely that combined mutations of multiple members of the MBD protein family could lead to a stronger phenotype, more closely resembling that of *met1*.

Our results do not exclude the possibility that MBD5 and MBD6 could play important roles in other rare cell types. For instance, the central cell of the female gametophyte shares several features with the pollen VN: decondensed and partially demethylated chromatin due, in part, to DME’s demethylation activity.^55,56^ While thousands of pollen grains are present in each flower bud, only about 50 central cells exist at the right developmental stage. Therefore, the investigation of transcriptional changes occurring in central cells requires direct isolation of this rare cell type. The endosperm tissue, which originates from fertilization of the central cell, has decondensed chromatin as well, and it is the tissue in which imprinting occurs in *Arabidopsis*.^57^ Given that MBD5/6 are required for silencing of the imprinted gene *FWA* in pollen, it will be interesting to investigate their role in imprinting regulation in endosperm.

We found that other repressors that act downstream of DNA methylation, MOM1 and MORC, do not show the same VN-specific phenotype as MBD5/6. Consistent with this finding, mutations in these factors cause reactivation of TEs in non-reproductive tissues, such as seedlings.^52,54^ The observation that, in *mom1* and *morc*, which are mild mutants like *mbd5/6*, TE derepression is not enhanced in the pollen VN suggests that the tissue specificity of the *mbd5/6* transcriptional phenotype could be due to the specific mechanism of silencing of the methyl readers as opposed to the other repressors. MBD5/6 could be required for gene silencing only at accessible promoters by, for instance, preventing the transcriptional machinery from binding the DNA. On the other hand, if the silencing mechanism of MOM1 and MORC involves chromatin decompaction, as previously suggested for MORC proteins,^58^ this might explain why the MBD5/6 targets, which are already decompacted, are not derepressed in *mom1* and *morc* (Figure 5G).

Most of the *morc* DEGs were strongly derepressed in MN. Consistently, several members of the MORC family are most strongly expressed in these nuclei, while MOM1, for instance, has a broad expression pattern and has DEGs in all nucleus types (Figure S5M). MORC5 is highly expressed in GN1 (Figure S5M), but we observed only one upregulated DEG in that cluster (Figure S7). It is possible that MORC5 plays a role in GN that is not related to gene or TE repression. The strong transcriptional effect of MORC proteins in early male gametogenesis is reminiscent of the known role of some animal MORC family members in the male germline.^59–62^ However, plant MORC proteins are not required for male fertility, and we did not observe any pollen development defect in the *morc hextuple* mutant, thus leaving open the question of whether *Arabidopsis* MORCs play a role in pollen biology.

In addition to providing insights into silencing mechanisms, this work contributes a comprehensive snRNA-seq dataset capturing the developing male gametophyte nuclei throughout development, from early microspores to mature pollen. This constitutes an important resource that can be further explored to gain insights into pollen biology and development. This dataset can be easily explored with an interactive website (https://singlecell.mcdb.ucla.edu/snRNAseq_pollen/), and the pollen transcriptome reannotations and codes used to generate them are publicly available (https://github.com/clp90/mbd56_pollen/).

### Limitations of the study

While our study identifies an important role for MBD5 and MBD6 in the pollen VN, we cannot exclude the possibility that these proteins could play important functions in other rare cell types that we have not directly inspected, such as the central cell or the endosperm.

## STAR★METHODS

### RESOURCE AVAILABILITY

#### Lead contact

Further information and requests for resources and reagents should be directed to and will be fulfilled by the lead contact, Steve Jacobsen (jacobsen@ucla.edu).

#### Materials availability

Seeds for all Arabidopsis lines generated in this study are available upon request to the lead contact.

#### Data and code availability

All sequencing data have been deposited at GEO and are publicly available as of the date of publication. The accession number is listed in the key resources table (GEO: GSE202422). The snRNA-seq data can be freely inspected in an interactive website (https://singlecell.mcdb.ucla.edu/snRNAseq_pollen/).

Microscopy data reported in this paper will be shared by the lead contact upon request.

This paper analyses existing, publicly available data. These accession numbers for the datasets are listed in the key resources table.

The pollen transcriptome reannotations and the codes used to generate them have been deposited in github (https://github.com/clp90/mbd56_pollen) and are publicly available as of the date of publication. DOIs are listed in the key resources table. All other codes used in this study are available upon request.

Any additional information required to reanalyze the data reported in this paper is available from the lead contact upon request.

### EXPERIMENTAL MODEL AND SUBJECT DETAILS

All plants used in this study were in the Columbia-0 ecotype (Col-0) and were grown on soil in a greenhouse under long-day conditions (16h light/8h dark). The experiments performed on seedlings were done after growing the plants on 1/2 MS medium plates under constant light.

The following mutant lines were obtained from Arabidopsis Biological Resource Center (ABRC) or previously generated as indicated in the *mbd5* (SAILseq_750_A09.1), *mbd6* (SALK_043927), *sln* (SALK_090484),^22^ *met1–3* (CS16394), *nrpe1–11*, *drm1 drm2*,^66^ *cmt2 cmt3*,^28^ *ddm1–2*,^64^ *mom1–3* (SALK_141293), *morc* hextuple consisting of *morc1–2* (SAIL_893_B06) *morc2–1* (SALK_072774C) *morc4–1* (GK-249F08) *morc5–1* (SALK_049050C) *morc6–3* (GABI_599B06) and *morc7–1* (SALK_051729).^54^ The *h1.1–1 h1.2–1* double mutant (referred to as *h1*) consists of SALK_128430 and GABI_406H11.^65^ The *mbd5/6* T-DNA double mutant and the *mbd5/6* CRISPR-1 mutant were previously described.^22^ The *mbd5/6* CRISPR-2 mutant was generated via CRISPR/Cas9 in the Col0 background as described in the next section.

The *mbd5/6 h1* and *sln h1* mutants were generated by crossing. The seedlings used for RNA-seq of *sln, h1, sln h1* and wild-type controls where F3 plants derived from individual F2 segregants of the cross with the indicated genotypes. The *mbd5/6 h1* experiment instead was performed with Col0, *mbd5/6*, and *h1* controls grown from the original batch of seeds used for the cross.

### METHOD DETAILS

#### Generation of *mbd5/6* CRISPR-2 line

The *mbd5/6* CRISPR-2 line was generated using the previously published pYAO::hSpCas9 system.^87^ We cloned two different guides for MBD5 (G1: ACCGGAGAACCCGGCTACTC, G2: GAAATCTAAAGTTCGATGTG) and two different guides for MBD6 (G1: TTCCGGTGCCACAGCTGGTT, G2: ATATGTTAGGGTTACTTAAT) performing four sequential cloning steps. Each guide was cloned in the AtU6–26-sgRNA cassette by overlapping PCR with primer tails containing the guide sequence. The PCR products were cloned into the SpeI site of the pYAO::hSpCas9 destination plasmid in four steps of SpeI digestion followed by In-Fusion (Takara). The final vector was electroporated into AGLO agrobacteria and transformed in Col0 plants by agrobacterium-mediated floral dipping. The transgenic lines were genotyped both by PCR amplification of the area surrounding the two guides to detect large deletions, and by Sanger sequencing to detect indels. The *mbd5/6* CRISPR-2 line was isolated in the T3 generation and was confirmed to have segregated out the pYAO::hSpCas9 transgene. This line has a homozygous G insertion at the MBD6 guide 1 region, which causes a frameshift and a premature STOP codon. The MBD5 mutation instead is a large inversion encompassing the entire region between the guide 1 and guide 2, which causes a frameshift and a premature STOP codon.

#### Generation of transgenic lines

The *pFWA::GUS* transgenic lines were generated with a published expression vector.^27^ The vector was electroporated into AGL0 agrobacteria that were used for plant transformation by agrobacterium-mediated floral dipping. Transformants were selected 1/2 MS medium plates with kanamycin, and the kanamycin resistant T1 plants were transplanted on soil. The GUS staining and imaging was performed in the T1 generation.

#### GUS staining and imaging

The experiment was performed in multiple batches with a total of least 10 individual *pFWA:*:GUS T1 transgenic lines for each genotype (Col0 and *mbd5/6* T-DNA). The two genotypes were always processed side by side. One inflorescence from each plant was clipped and placed in cold acetone on ice. The samples were transferred to −20°C for 30 min. After the incubation, the inflorescences were washed two times with room temperature water, and then transferred in GUS staining solution (1 mL of 0.1 M X-Gluc, 1.71 mL of 1 M Na_2_H, 0.79 mL of 1 M NaH_2_, 2.5 mL of 0.1 M potassium ferrocyanide, 2.5 mL of 0.1 M potassium ferricyanide, 100 ul of Triton X100, water up to 50 mL). The samples were vacuum infiltrated for about 10 min and then incubated at 37°C for about 4–5 h. The reaction was stopped by transferring the samples to 70% ethanol, and the inflorescences were kept in 70% ethanol for 3 days with gentle shaking, changing the ethanol solution every day. Next, the samples were washed with water and then incubated in ClearSee solution for one day.^88^ Samples were imaged with a ZEISS Stemi 508 Stereo Microscope with Axiocam 208 Color. The images were processed with ZEISS ZEN lite.

For high magnification imaging of GUS-stained developing pollen grains, five to seven inflorescences were harvested in 500–700 μL of 0.1 M mannitol solution, in 1.5 mL tubes. The flower buds were gently disrupted with a pestle to release the spores in solution. The solution was filtered over 80 μm nylon mesh two times, and then centrifuged over a cushion of 500 μL of 45% Percoll in 0.1 M mannitol, for 10 min at 800 g. The pellet containing the cleaned mixed stage spores was resuspended in 200 μL of GUS staining solution and incubated at 37°C for about 4–5 h. Next, the samples were collected by brief centrifugation at 500 g, and the pellets were resuspended in 10 μL of DAPI buffer (0.4 μg/mL DAPI solution in 0.1 M sodium phosphate buffer, 10 mM EDTA–disodium salt and 0.1% Triton X-100, pH 7.0).^89^ After a 5 min incubation, 5 μL of each sample were transferred to a microscopy slide, covered with a glass coverslip and sealed with nail polish. Samples were imaged immediately with an Axio Imager.D2 upright microscope.

#### Dissection of anthers for RNA-seq

Anthers were manually dissected from 20 individual flower buds (stage 10–12) for each sample (two samples per genotype obtained from different plants). Dissected anthers were placed immediately in 350 μL of ice-cold RLT buffer from the RNeasy micro kit (Qiagen, 74004) keeping the tube on ice. For each flower bud, all other remaining tissues were placed in a separate tube with 350 μL of ice-cold RLTbuffer, to prepare the “no-anthers” fraction. The tissues were disrupted on ice in the RLT buffer using a sterile pestle (Axygen, PES-15-B-SI). We then proceeded with RNA extraction using the RNeasy micro kit (Qiagen, 74004) starting from step 4 (addition of one volume of 70% ethanol). In-column DNAse digestion was carried on following manufacturer’s instructions. RNA-seq libraries were generated using the TruSeq Stranded mRNA Library Prep Kit (Illumina) with the standard workflow, starting with 300–400 ng of RNA.

#### Bulk RNA-seq of seedlings, flower buds, and mature pollen

##### Samples

Biological triplicates were generated for each genotype.

Seedlings RNA-seq was done by harvesting ~10–15 14 days old seedlings from ½ MS plates and flash freezing them in liquid nitrogen (replicated were grown in separate plates).

Unopened flower bud RNA-seq was done by harvesting one inflorescence (excluding open flowers) from an individual plant for each sample and freezing it immediately in liquid nitrogen.

Mature pollen RNA-seq was performed by harvesting the pollen with the previously described vacuum method.^90^ Briefly, about 150 plants were grown for each genotype. We assembled an in-house filtering system on a vacuum cleaner to place three different nylon meshes at the end of the tube in this order: 80 μm, 31 μm, 7 μm. Using this system, we aspirated the pollen from the plants. The 80 μm and 31 μm filters block the flower parts such as petals, the soil and other particulate, while the pollen accumulates on the 7 μm mesh. We then collected the pollen from the 7 μm nylon mesh using a pipette with 0.1 M mannitol and transferred it into a 1.5 mL tube. The solution was centrifugated for 5 min at 500 g. The supernatant was removed, and the pollen pellet was frozen in liquid nitrogen and stored at −80°C. This procedure was repeated every 2/3 days to obtain replicates for each genotype.

In the case of *met1*, given the difficulty to obtain high numbers of homozygous mutant plants, we used a different protocol to purify mature pollen from a smaller number of plants. We harvested ~500 μL of open flowers from *met1* and Col0 control plants in 2-mL protein low bind tubes (Eppendorf). We then added 800 μL of Galbraith buffer (45 mM MgCl_2_, 30 mM C_6_H_5_Na_3_O_7_.2H_2_O [Trisodium citrate dihydrate], 20 mM MOPS, 0.1% [v/v] Triton X-100, pH 7), and vortexed the samples for 3 min at max speed to release the pollen from the anthers. The suspension was filtered with an 80 μm nylon mesh into a new 1.5 mL tube. The procedure was repeated one more time with the same flowers to increase the yield of pollen. The two aliquots of filtered pollen suspension were combined and centrifuged for 5 min at 800 g, 4°C. The pollen pellet was frozen in liquid nitrogen and stored at −80°C. This procedure was repeated every 2/3 days to obtain replicates.

#### RNA extraction and library preparation

Frozen samples were disrupted with a tissue grinder and RNA extraction was performed with the Zymo Direct-zol RNA MiniPrep kit (Zymo Research), or in the case of the pollen samples with the QIAGEN RNeasy Mini kit (Qiagen). In both cases, the in-column DNase digestion was performed.

RNA-seq libraries were generated using the TruSeq Stranded mRNA Library Prep Kit (Illumina), following the manufacturer’s instructions and starting with 1 μg of RNA as input for flowers and seedlings, and 300–500 ng for the pollen samples.

#### Single-nucleus RNA-seq of developing male gametophytes

The protocol for isolation of mixed-stage male gametophytes was developed starting from a published protocol.^91^ We processed 2 or 3 genotypes at the time. Unless otherwise specified, each buffer was freshly supplemented with 70 mM 2-Mercaptoethanol and cOmplete^™^ Protease Inhibitor Cocktail (Sigma). For each genotype, we harvested on ice about 5 mL of unopened flower buds including one open flower for each inflorescence. The spores were released from the buds in a prechilled mortar on ice, using 5 mL of 0.1 M mannitol, by gently tapping with a prechilled pestle for 1–2 min. The suspension was transferred to a 50 mL conical tube, and an additional 10 mL of 0.1 M mannitol were used to rinse the mortar. The tube was then vortexed intermittently for 30 s to release the spores, and the suspension was filtered through a 100 μm nylon membrane to remove the tissue. An additional 5 mL of 0.1 M mannitol were used to rinse the tube and poured over the same filter, obtaining a total of 20 mL of suspension for each sample. The spores were further filtered twice through a 60 μm nylon mesh and then divided into two 15-mL glass tubes. The tubes were spun with a Sorvall Lynx 4000 Centrifuge (Thermo Scientific) with TH13–6×50 swing-out rotor, for 10 min at 900 g, 4°C, acceleration speed 5, braking speed 8. The supernatant was carefully removed, and the pellets were resuspended in 1 mL of ice-cold 0.1 M mannitol. Each aliquot was transferred to a new tube and layered above 3 mL of 20% Percoll (diluted in 0.1 M Mannitol). The samples were centrifuged at 450 g for 10 min at 4°C, with acceleration speed 5 and braking speed 8. The two pellets from the same genotype were combined with 2 mL of 0.1 M mannitol, and the centrifugation over 20% Percoll was repeated two times to clean the spores further. The purified mixed spores were then transferred to a 1.5 mL tube and inspected under the microscope.

The protocol for nuclei extraction and purification was adapted from.^31^ The spores were pelleted via 5 min centrifugation at 500 g, 4°C. The pellets were resuspended in 800 μL of Galbraith buffer supplemented with 70 mM 2-Mercaptoethanol (45 mM MgCl_2_, 30 mM C_6_H_5_Na_3_O_7_.2H_2_O [Trisodium citrate dihydrate], 20 mM MOPS, 0.1% [v/v] Triton X-100, pH 7). The suspension was transferred to a 1.5 mL tube containing 100 μL of acid-washed 0.5 mm glass beads (Sigma). To break the pollen walls, the samples were vortexed at max speed for 2 min total in a cold room with the following vortexing protocol: 7 s vortex, 3 s invert for the first minute followed by 7 s vortex, 2 s invert for the second minute. We then filtered the nuclei by briefly spinning them over a 10 μm cellTrics filter placed in a clean 1.5 mL tube. 400 μL of Galbraith buffer were used to rinse the beads, and added to the cellTrics filter. While keeping the flowthrough on ice, the unbroken pollen grains that remained on the filter were collected by pipetting with 800 μL of Galbraith buffer and were placed back in the tube with the glass beads. An additional round of vortexing and filtering was performed as before, to increase the nuclei yield. The nuclei in the two tubes were then spun down for 5 min at 500 g, 4°C, and the pellets were resuspended by gentle pipetting with 50 μL of CyStain UV Precise P - Nuclei Extraction Buffer (Sysmex, 05–5002-P02). To stain the nuclei, we then added 400 μL of CyStain UV Precise P – Staining Buffer (Sysmex, 05–5002-P01) and we supplemented the sample with Protector RNase Inhibitor (Sigma) to a final concentration of 0.2 U/μL. The samples were passed over the filter of a FACS tube (Falcon 352235) and immediately sorted. Sorting was done with a BD FACS ARIAII instrument equipped with a 355nm UV laser, using the 70μm nozzle. The gating strategy is shown in Figures S5A–S5C. For each sample, we sorted 40,000–60,000 nuclei in 500 μL of Nuclei wash buffer (2% BSA in 1X PBS) supplemented with Protector RNase Inhibitor (Sigma) to a final concentration of 0.2 U/μL. The sorted nuclei were pelleted by centrifugation for 5 min at 500 g, 4°C. The pellet was resuspended in 20–25 μL of buffer and the entire sample was used as input for the 10x Genomics Chromium Single Cell 3^′^ Reagent Kit v3. The subsequent steps were performed according to the manufacturer’s instructions.

### QUANTIFICATION AND STATISTICAL ANALYSIS

#### Analysis of BS-seq

The BS-seq data present in this paper was reanalyzed from the following deposited datasets. Wild-type flower BS-seq: GSM5026060 and GSM5026061 merged replicates.^22^ Wild-type sorted SN: GSM952445.^15^ Wild-type sorted VN: GSM952447.^15^ Raw reads were trimmed with TrimGalore (Babraham Institute) and mapped to the TAIR10 genome with Bismark.^67^ Bismark was also used to obtain the methylation percentages for each cytosine and to generate the per-position DNA methylation tracks. The quantification of the average methylation percentage at promoters was calculated with bedtoolsmap^68^ with the option “*mean*”. Promoters were defined as a 600 bp region surrounding the TSS.

#### Analysis of bulk RNA-seq

The bulk RNA-seq data was analyzed as previously described.^22^ The RNA-seq reads were filtered based on quality score and trimmed to remove Illumina adapters using Trim Galore (Babraham Institute). The filtered reads were mapped to the Arabidopsis reference genome (TAIR10) using STAR,^69^ allowing 5% of mismatches (-outFilterMismatchNoverReadLmax 0.05) and unique mapping (–outFilterMultimapNmax 1). PCR duplicates were removed using MarkDuplicates from the Picard Tools suite. Coverage tracks for visualization in the genome browser were generated using Deeptools 3.0.2 bamCoverage with the options –normalizeUsing RPKM and –binSize 10.^70^ The number of reads mapping to genes or transposable elements were determined using HTseq^71^ with the option –mode = union. We used as reference the transcriptome annotations generated as described in the paragraph “pollen transcriptome reannotation”, and all transcripts were analyzed together in the DEG analysis (genes, TEs, and other undefined non-coding transcripts). The HTseq gene counts were used to perform the differential gene expression analysis using the R package DEseq2^92^ with a cutoff for significance of padj <0.05 and |log2FC|>0.5. The transcripts per million (TPM) values were estimated using Kallisto version 0.46.0.^72^ Figures were generated using the R package ggplot. The heatmaps of RNA-seq and methylation data were made with the R package *ComplexHeatmap*.^93^

The TE family analysis was done using the package *TEtranscripts*^73^ Reads were mapped with STAR allowing multimapping up to 100 hits (–outFilterMultimapNmax 100 and –winAnchorMultimapNmax 100). TEtranscripts was run with default options (–mode multi). Significantly upregulated TE families were defined as the ones with adjusted p value < 0.05.

The gene ontology (GO) analysis of the *met1* DEGs was done with the R package *clusterProfiler*,^94^ running *enrichGO* with the following parameters: ont = “all”, pvalueCutoff = 0.05, qvalueCutoff = 0.10. We used as reference genes the list of all genes with base-Mean>2 (from the DEseq2 table).

The following RNA-seq datasets were downloaded from GEO and reanalyzed as described above: wild-type VN (GSM4700179, GSM4700180, GSM4700181),^19^ wild-type sperm (GSM4700188, GSM4700189, GSM4700190),^44^ wild-type and *mbd5/6* flower buds (GSM5026083 to GSM5026091),^22^ wild-type*, sln* and *met1* flower buds (GSM5026092 to GSM5026094 and GSM5026098 to GSM5026103),^22^ wild-type and *met1* seedlings (GSM938342, GSM938343, GSM938348, GSM938349).^63^

The curated list of “pollen-tube related genes” that are demethylated by DME, used in Figure 6B, includes: AT1G66235, AT2G19480, AT1G44120, AT2G16586, AT2G14260, AT5G28470, AT1G35540, AT3G30720, AT1G74800, AT2G16015, AT2G22055/RALFL15, AT2G07040/PRK2, novel_Chr1_coding_102/RKF2/AT1G19090, novel_Chr2_coding_224/PLOU/AT2G16030.

#### Pollen transcriptome reannotation

All Pollen RNA-seq libraries (27 libraries total) were filtered and trimmed as described in previous section (“analysis of bulk RNA-seq”), and aligned to the Arabidopsis reference genome (TAIR10) using STAR.^69^ STAR genome indexes were made using the Araport11 annotations from Cheng et al.^95^ BAM files for the 27 pollen RNA-seq samples were pooled into a single BAM file using samtools merge, with a total of approximately 450M reads in the pool. Note all libraries were made using the Truseq stranded RNA kit and shared the same ‘strandedness’. Transcripts were assembled from this BAM file using four different programs: Trinity v2.13.2,^74^ Cufflinks v.2.2.1,^75^ CLASS2 v.2.1.7^76^ and StringTie v.2.1.6.^77^ When annotation information could also be provided to guide assembly (Trinity, Cufflinks, and StringTie), Araport11 was again provided. Trinity was run with options –genome_guided_bam pooled.bam -genome_guided_max_intron 2000 -jaccard_clip -SS_lib_type RF. The resulting fasta file was converted to GTF using gmap version 2021–08–25.^78^ Cufflinks was run with options –g araport11.gtf –I 5000 –library-type fr-firststrand–max-bundle-length 30000 –min-intron-length 15 –overlap-radius 1. CLASS2 was run with default options, and StringTie with options –G araport11.gtf –rf -m40 –g 1. Additionally, junctions were detected using Portcullis v.1.2.2^79^ with options –orientation FR –strandedness firststrand –max_length 2000. Assembled transcripts from each of these sources were combined and best transcripts were selected using Mikado v.2.3.2.^80^ Mikado is run in 4 parts: (1) configure with options –strand-specific and providing Portcullis predictions to –junctions, (2) prepare with default options; this step pools predicted transcripts from all input sources (3) serialise with –orfs = ORFs predicted by prodigal v.2.6.3^81^ with options –g1 –f gff and –xml blastx output from BLASTX v.2.11.0 with options –max_target_seqs 5 –outfmt “6qseqid sseqid pident length mismatch gapopen qstart qend sstart send evalue bitscore ppos btop”, and (4) pick, with options –scoring-file plant.yaml –no-purge (plant.yaml from Mikado Github). Mikado selections were further refined using custom python script mikado_refine.py (available from Github at https://github.com/clp90/mbd56_pollen), which incorporates additional RNA-seq coverage information and a few other selection parameters, with default options. To simplify our analysis, mikado_refine.py outputs a single ‘best’ representative annotation for each gene. Final updated annotations and codes used to generate them are available from Github at https://github.com/clp90/mbd56_pollen.

#### Analysis of ATAC-seq

The bigWig track files for the wild-type VN, GN, and SN ATAC-seq datasets were downloaded from GEO (GSM4699541 to GSM4699544, and GSM5027098 GSM5027100).^19^ The ATAC-seq enrichment at promoters was obtained with deeptools multiBigwigSummary with the –BED option and –outRawCounts.^70^ The promoter regions were defined as windows from 300 bp before the TSS until 300 bp after the TSS.

#### Analysis of snRNA-seq

##### Preprocessing

Cell Ranger 6.1.1 software (10X genomics) was used to process the raw data. The reads were aligned to the Arabidopsis reference annotations generated as explained in “Pollen transcriptome reannotations”. For each individual sample, we then removed the ambient RNA using SoupX with standard settings.^82^ The data was then imported in Seurat 4.0.4^83^ removing cells in which less than 300 genes were detected. The data was normalized with *NormalizeData* (normalization.method = “LogNormalize”, scale.factor = *10000*), and scaled with *ScaleData* with default settings. We then performed principal component analysis on all genes with *RunPCA* (npcs = 20) and dimensionality reduction with *RunUMAP* using the first 20 principal components (PCs). Next, we used DoubletFinder v3^84^ to identify doublets with the standard workflow based on the first 20 principal components, and we used *find.pK* to identify the optimal pK parameter for each sample. The pK values and the percentage of doublets removed for each sample are available in Table S3. Lastly, we removed nuclei with more than 5% of mitochondrial reads or more than 15% of chloroplast reads.

##### Integration and clustering

The different datasets were integrated with Seurat v4 *FindIntegrationAnchors* and *IntegrateData* using default settings (nfeatures = 2000). The integrated data was scaled with *ScaleData* with default settings, and PCA analysis was performed with *RunPCA* (npcs = 40). Dimensionality reduction was done with *RunUMAP* using the first 40 PCs. We then performed clustering analysis using *FindNeighbors* to calculate the k-nearest neighbors based on the first 40 PCs, and *FindClusters* with resolution = 0.3, using the Louvain algorithm (default). The list of numbers of cells per cluster for each sample is available in Table S3. We note that the relative number of cells per cluster was quite variable when comparing different wild-type datasets, likely because of the variability introduced while harvesting the inflorescences. Therefore, we think that this experimental approach cannot be used to determine how a given mutation can impact the speed of specific stages of pollen development, unless the phenotype is very strong such as in the case of *met1* (Figure 5A).

##### Cluster markers and gene ontology (GO) analysis

The cluster markers (available in Table S4) were obtained with the Seurat function *FindAllMarkers* ran on the Col0 samples only, using the options only.pos = TRUE and logfc.threshold = 0.1. This generated a non-stringent list of markers that were ranked by average log2FC. To perform the GO analysis, we selected the markers with p_val_adj <0.05 and avg_log2FC > 1, and we used as reference the list of all Arabidopsis genes included in our reference transcriptome. The analysis was done with the R package *clusterProfiler*^94^, running *enrichGO* with the following parameters: ont = “all”, pvalueCutoff = 0.05, qvalueCutoff = 0.10. The complete list of results is available in Table S6.

##### Pseudotime analysis

The VN pseudotime analysis was performed with Monocle3^35,86,85^ following the standard workflows. The Seurat object was subsetted to select the Col0 cells assigned to the VN clusters. The data was then converted into a Monocle3 cell_data_set object with the R package “*SeuratWrappers*”. The trajectory was constructed with the *learn_graph* function and the root was manually selected with the order_cells function (reduction_method = “UMAP”). To find the genes that vary as a function of pseudotime, we used the *graph_test* function with the option neighbor_graph = “principal_graph”, to test whether cells at similar positions on the trajectory have correlated expression. We then selected the genes with q_value <0.01 & morans_I > 0.1. The Seurat function *AverageExpression* with the option slot = “data” was used to obtain the average expression levels of those genes (log normalized), for all the cells belonging to a given pseudotime interval (binning of the pseudotime in 43 intervals). The average expression values were scaled by z-score, visualized in a heatmap and clustered with the R package *pheatmap* (using *hclust*). Gene ontology analysis was then performed on each group of genes as described in the paragraph “cluster markers and gene ontology (GO) analysis”.

##### Differential gene expression (DEG) analysis of snRNA-seq data

Given the well described challenges of performing DEG analysis with single cell RNA-seq data, we tested several different approaches, including both pseudobulk and single cell methods as suggested in the literature.^96^ The pseudobulk methods that take into account biological replicates are considered more robust; however, when we used either pseudobulk or single cell methods to identify the *mbd5/6* DEGs, we observed a large number of DEGs that corresponded to highly expressed and unmethylated genes, and therefore don’t have the typical features of MBD5/6 targets. Thus, we decided to take advantage of the 6 independent wild-type replicates that we generated on different days to compile a list of “noise DEGs” including genes that are called as differentially expressed when comparing wild-types with each other. We then subtracted these genes from the lists of DEGs obtained for each mutant compared with its matched wild-type (Figures S6A and S6B). Without filtering the “noise DEGs”, most of the *mbd5/6* upDEGs are unmethylated genes that are not upregulated in mature pollen bulk RNA-seq (Figure S6C), while after filtering, almost all the DEGs have promoter methylation and are upregulated in bulk RNA-seq as well (Figure 4C). This observation gave us confidence in the validity of our method. We do, however, acknowledge that this approach is very stringent and could bias the analysis towards genes that are lowly or not expressed in wild-type. For the purpose of this study, however, this is not a concern because we are investigating derepression mutants, which mostly affect loci that are lowly or not expressed in wild-type. In the next paragraph we explain in more detail the methods used to analyze and plot the data.

The DEG analysis was done by running on each individual cluster the Seurat function *FindMarkers* with “wilcox” as statistical method. All transcripts were considered together for the DEG analysis (genes, TEs, and other non-coding transcripts). Each mutant was compared with its matched wild-type control (processed on the same day). We also compared all the wild-type samples to each other, and we selected the significant DEGs (p_val_adj<0.05) to obtain the list of “noise DEGs”, which were filtered out of each list of mutant DEGs. Downstream analyses were done using the filtered mutant DEGs that had an avg_log2FC < −0.25 or >0.25. For some analyses we grouped the DEGs in the following categories: “MN DEGs” are the union of MN1, MN2, and MN3 DEGs. “Early VN DEGs” are the union of VN1, VN2, and VN3. “Late VN DEGs” are the union of VN4 and VN5 DEGs.

To visualize the data, we grouped together nuclei clusters that were similar. The cluster groups were defined as follows: MN1, MN2, and MN3 were combined into the “MN” group, VN1, VN2 and VN3 were combined into the “early VN” group, VN4 and VN5 in the “late VN” group, GN1 and GN2 in the “GN” group. The heatmaps were generated by first extracting from the Seurat object the average expression values per cluster group, using the Seurat function *AverageExpression* with the option slot = “data”. These values were then scaled by calculating for each gene the z-score across all columns. To combine the snRNA-seq, BS-seq, and bulk RNA-seq in a heatmap we used the R package *ComplexHeatmap*. The boxplots of log2FC were generated with the R package *ggplo*t, using the average log2FC values for each cluster group obtained using the Seurat function *FindMarkers*.

### ADDITIONAL RESOURCES

The snRNA-seq can be inspected with an interactive website: https://singlecell.mcdb.ucla.edu/snRNAseq_pollen/.

## Supplementary Material

1

2

3

4

5

6

7

8

## Figures and Tables

**KEY RESOURCES TABLE:** 

REAGENT or RESOURCE	SOURCE	IDENTIFIER

Bacterial and virus strains		

Escherichia coli NEB10-beta	New England Biolabs	C3019H
Agrobacterium tumefaciens AGL0	N/A	N/A

Chemicals, peptides, and recombinant proteins		

Complete EDTA-free Protease Inhibitor	Roche	5056489001
PMSF	SIGMA	P7626
2-Mercaptoethanol	Thermo Fisher	CAS:60-24-2
D-Mannitol	SIGMA	M1902
Percoll	GE Healthcare	17-0891-01
BSA	SIGMA	A7906
Protector RNase Inhibitor	SIGMA	3335402001
MgCl2	Fisher	CAS:7791-18-6
Trisodium citrate dehydrate	SIGMA	C8532
Triton X100	SIGMA	SKU:X100
MOPS	SIGMA	SKU:69947
SpeI-HF	NEB	R3133
CyStain UV Precise P - Nuclei Extraction Buffer	Sysmex	05-5002-P02
CyStain UV Precise P - Staining Buffer	Sysmex	05-5002-P01

Critical commercial assays		

Direct-zol RNA miniprep kit	ZYMO	R2052
RNeasy Micro Kit	Qiagen	74004
TruSeq Stranded mRNA Library Prep kit	Illumina	20020594
Chromium Next GEM Single Cell 3’ Kit v3.1	10X genomics	PN-1000268

Deposited data		

Bulk RNA-seq and snRNA-seq	This study	GEO: GSE202422, https://singlecell.mcdb.ucla.edu/snRNAseq_pollen/
RNA-seq wild-type VN	Borg et al.^19^	GEO: GSM4700179, GEO: GSM4700180, GEO: GSM4700181
RNA-seq wild-type sperm	Borg et al.^19^	GEO: GSM4700188, GEO: GSM4700189, GEO: GSM4700190
RNA-seq mbd5/6 flower buds	Ichino et al.^22^	GEO: GSM5026083 to GSM5026091
RNA-seq met1 and sln flower buds	Ichino et al.^22^	GEO: GSM5026092 to GSM5026094 and GEO: GSM5026098 to GSM5026103
RNA-seq met1 seedlings	Stroud et al.^63^	GEO: GSM938342, GEO: GSM938343, GEO: GSM938348, GEO: GSM938349
Wild-type flower BS-seq	Ichino et al.^22^	GEO: GSM5026060 and GSM5026061
Wild-type BS-seq sorted SN and VN	Ibarra et al.^15^	GEO: GSM952445 and GSM952447
ATAC-seq VN, GN and SN	Borg et al.^19^	GEO: GSM4699541 to GSM4699544, and GEO: GSM5027098 GSM5027100

Experimental models: Organisms/strains		

Col-0	Jacobsen lab	NA
*mbd5*	Ichino et al.^22^	SAILseq_750_A09.1
*mbd6*	Ichino et al.^22^	SALK_043927
mbd5/6 T-DNA	Ichino et al.^22^	SAILseq_750_A09.1 SALK_043927
mbd5/6 CRISPR1	Ichino et al.^22^	mbd5/6 CRISPR1
mbd5/6 CRISPR2	This paper	mbd5/6 CRISPR2
Sln	Ichino et al.^22^	SALK_090484
*met1-3*	Jacobsen lab	CS16394
*ddm1-2*	Vongs et al.^64^	ddm1-2
*mom1-3*	Jacobsen lab	SALK_141293
*more* hextuple	Harris et al.^54^	*more1-2* (SAIL_893_B06) *more2-1* (SALK_072774C) *more4-1* (GK-249F08) *more5-1* (SALK_049050C) *more6-3* (GABI_599B06) and *more7-1* (SALK_051729)
h1	Zemach et al.^65^	*h1.1-1 h1.2-1* (SALK_128430 and GABI_406H11)
*mbd5/6 h1*	This paper	SAILseq_750_A09.1 SALK_043927 SALK_128430 GABI_406H11
*sln h1*	This paper	SALK_090484 SALK_128430 GABI_406H11
nrpe1-11	N/A	SALK_029919
drm1 drm2	Chan et al.^66^	drm1-2 drm2-2
cmt2 cmt3	Stroud et al.^28^	cmt2-7 cmt3

Recombinant DNA		

pFWA::GUS	Ikeda et al.^27^	N/A

Software and algorithms		

TrimGalore	Babraham Institute	N/A
Bismark	Krueger and Andrews^67^	N/A
BEDTools	Quinlan and Hall^68^	N/A
STAR	Dobin et al.^69^	N/A
MarkDuplicates	Picard Tools	N/A
Deeptools 3.0.2	Ramírez et al.^70^	N/A
HTseq	Anders et al.^71^	N/A
DEseq2	Love et al.^71^	N/A
Kallisto	Bray et al.^72^	N/A
*TEtranseripts*	Jin et al.^73^	N/A
Trinity v2.13.2	Haas et al.^74^	N/A
Cufflinks v.2.2.1	Trapnell et al.^75^	N/A
CLASS2 v.2.1.7	Song et al.^76^	N/A
StringTie v.2.1.6	Pertea et al.^77^	N/A
gmap	Wu and Watanabe^78^	N/A
Portcullis v.1.2.2	Mapleson et al.^79^	N/A
Mikado v.2.3.2	Venturini et al.^80^	N/A
prodigal v.2.6.3	Hyatt et al.^81^	N/A
Cell Ranger 6.1.1 software	10X genomics	N/A
SoupX	Young and Behjati^82^	N/A
Seurat 4.0.4	Hao et al.^83^	N/A
DoubletFinder v3	McGinnis et al.^84^	N/A
Monocle3	Cao et al.^35^; Qiu et al.^85^; Trapnell et al.^86^	N/A
Code for pollen transcriptome reannotation	This study	https://github.com/clp90/mbd56_pollen

## Highlights

- snRNA-seq reveals transcriptional profiles of developing pollen nuclei
- Loss of silencing in *mbd5/6* and *sln* occurs in pollen vegetative nuclei
- *met1*, *ddm1*, *mom1*, and *morc* show loss of silencing in all pollen nucleus types
- Chromatin decompaction via H1 mutations enhances the *mbd5/6* and *sln* derepression
