# Supplementary material for: Single-nucleus RNA-seq reveals that MBD5, MBD6, and SILENZIO maintain silencing in the vegetative cell of developing pollen
Source: Cell Rep. Author manuscript; Available in PMC 2022 Dec 21. (PMC9770095; doi:10.1016/j.celrep.2022.111699)
Supplement: 1 [file NIHMS1852532-supplement-1.pdf]

Cell Reports, Volume *41*

Supplemental information

Single-nucleus RNA-seq reveals that MBD5, MBD6, and SILENZIO maintain silencing in the vegetative cell of developing pollen

Lucia Ichino, Colette L. Picard, Jaewon Yun, Meera Chotai, Shuya Wang, Evan K. Lin, Ranjith K. Papareddy, Yan Xue, and Steven E. Jacobsen

Supplementary figures

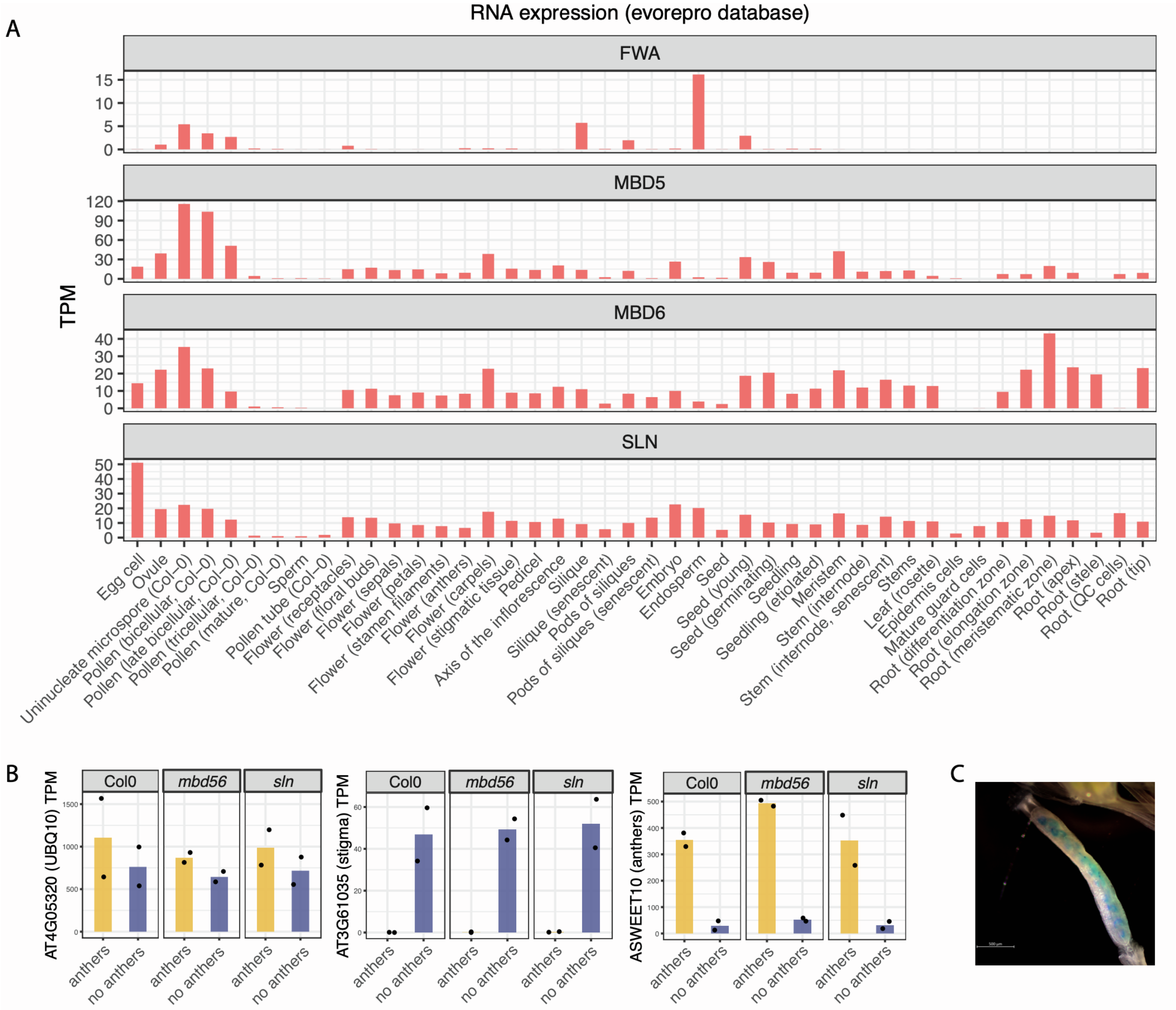

Figure S1 – related to Figure 1: Tissue specificity of *FWA* expression.

A) Barplots of average expression for the indicated genes (transcripts per million [TPM]) across a panel of tissues. The data was downloaded from https://evorepro.sbs.ntu.edu.sg and re-plotted. B) RNA-seq data of dissected flower buds (dissection strategy shown in Figure 1A). Shown are the average TPM values for a constitutively expressed gene (*POLYUBIQUITIN 10*), a gene enriched in stigmatic tissue (AT3G61035), and an anthers marker (*ASWEET10*). The dots indicate individual replicates. C) Representative image of the endogenous expression of *FWA* in early developing seeds. The *pFWA::GUS* reporter line was GUS-stained and cleared with ClearSee [S1]. Scale bar: 500 µm.

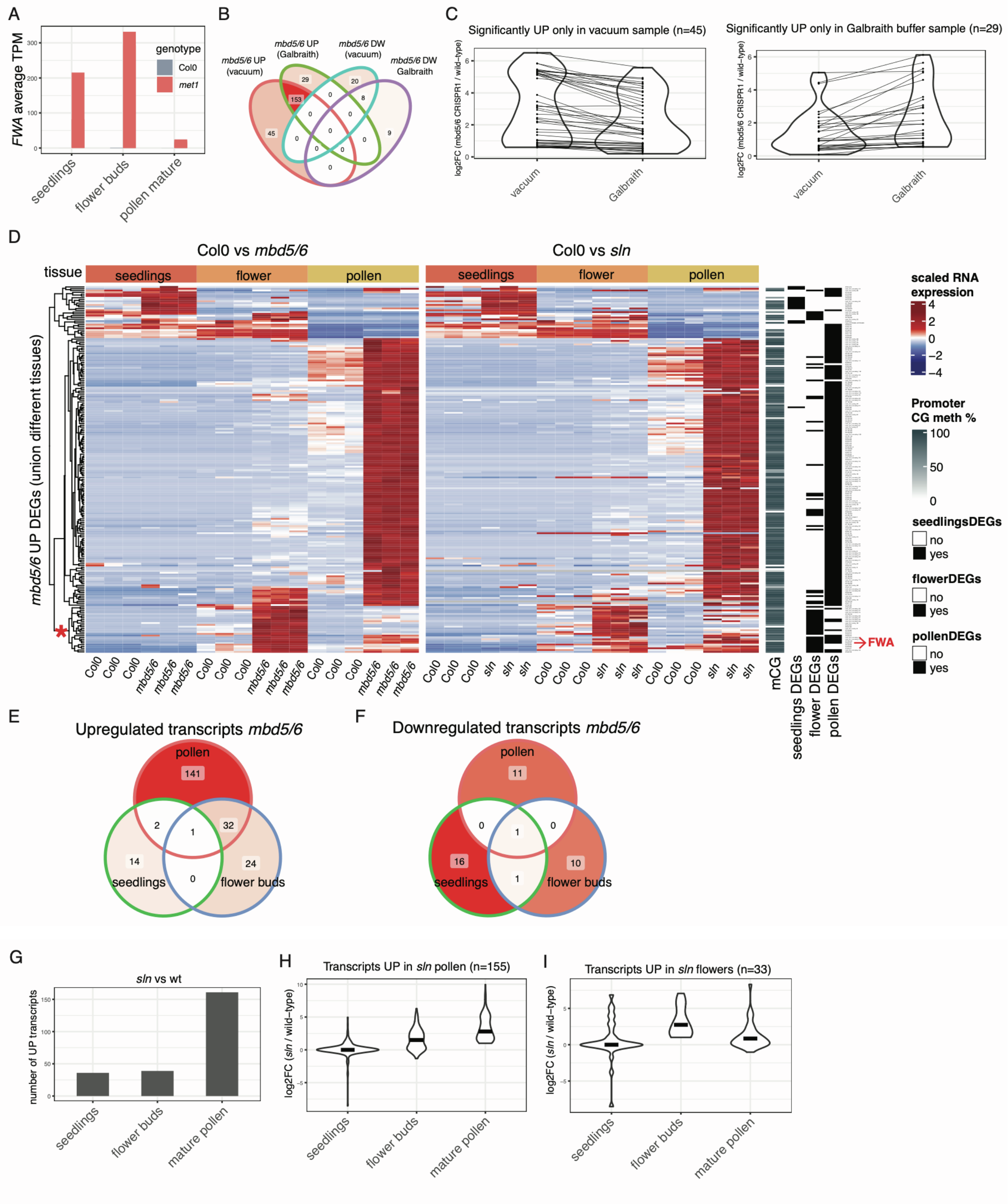

A
FWA average TPM
genotype
Col0
met1
seedlings
flower buds
pollen mature
B
mbd5/6 UP (vacuum)
mbd5/6 UP (Galbraith)
mbd5/6 DW (vacuum)
mbd5/6 DW Galbraith
C
Significantly UP only in vacuum sample (n=45)
Significantly UP only in Galbraith buffer sample (n=29)
log2FC (mbd5/6 CRISPR1 / wild−type)
vacuum
Galbraith
D
Col0 vs mbd5/6
Col0 vs sln
tissue
seedlings
flower
pollen
mbd5/6 UP DEGs (union different tissues)
mCG
seedlings DEGs
flower DEGs
pollen DEGs
FWA
scaled RNA expression
Promoter CG meth %
seedlingsDEGs
flowerDEGs
pollenDEGs
no
yes
E
Upregulated transcripts mbd5/6
pollen
seedlings
flower buds
F
Downregulated transcripts mbd5/6
pollen
seedlings
flower buds
G
sln vs wt
number of UP transcripts
seedlings
flower buds
mature pollen
H
Transcripts UP in sln pollen (n=155)
log2FC (sln / wild−type)
seedlings
flower buds
mature pollen
I
Transcripts UP in sln flowers (n=33)
log2FC (sln / wild−type)
seedlings
flower buds
mature pollen

Figure S2 – related to Figure 2: Tissue specificity of the *mbd5/6* and *sln* transcriptional derepression phenotypes.

A) *FWA* average expression in the indicated tissues measured by RNA-seq. The *met1* seedlings data is from [S2] and the flower buds data is from [S3]. B) Venn diagram of overlaps between the upregulated DEGs detected in *mbd5/6* pollen extracted with two different methods: vacuum aspiration or Galbraith buffer (see Methods). C) Violin plots showing the differential expression level (log2FC) of the transcripts that were called as significantly upregulated in only one of the two methods (vacuum aspiration or Galbraith buffer). D) Heatmap showing the union of the *mbd5/6* upregulated transcripts in seedlings, flower buds and mature pollen (n=204). The RNA-seq data is shown as z-score of TPM, the methylation data is the average CG methylation percentage in flower buds, in a 600 bp window centered on the TSS. The row annotations on the right indicate whether each gene was called as significant *mbd5/6* DEG in each tissue. The red asterisk on the dendrogram indicates a group of genes that are more strongly upregulated in flowers than in pollen. This group includes *FWA*, as shown on the right. E,F) Venn diagrams of upregulated and downregulated transcripts in *mbd5/6* seedlings, flower buds, and mature pollen. G) Number of upregulated transcripts in *sln* vs Col0 (wild-type) in the indicated tissues. The *sln* flower buds RNA-seq is from [S3]. H,I) Distribution of the *sln* vs Col0 log2 fold-changes for the indicated transcripts in different tissues. Black bar indicates the median.

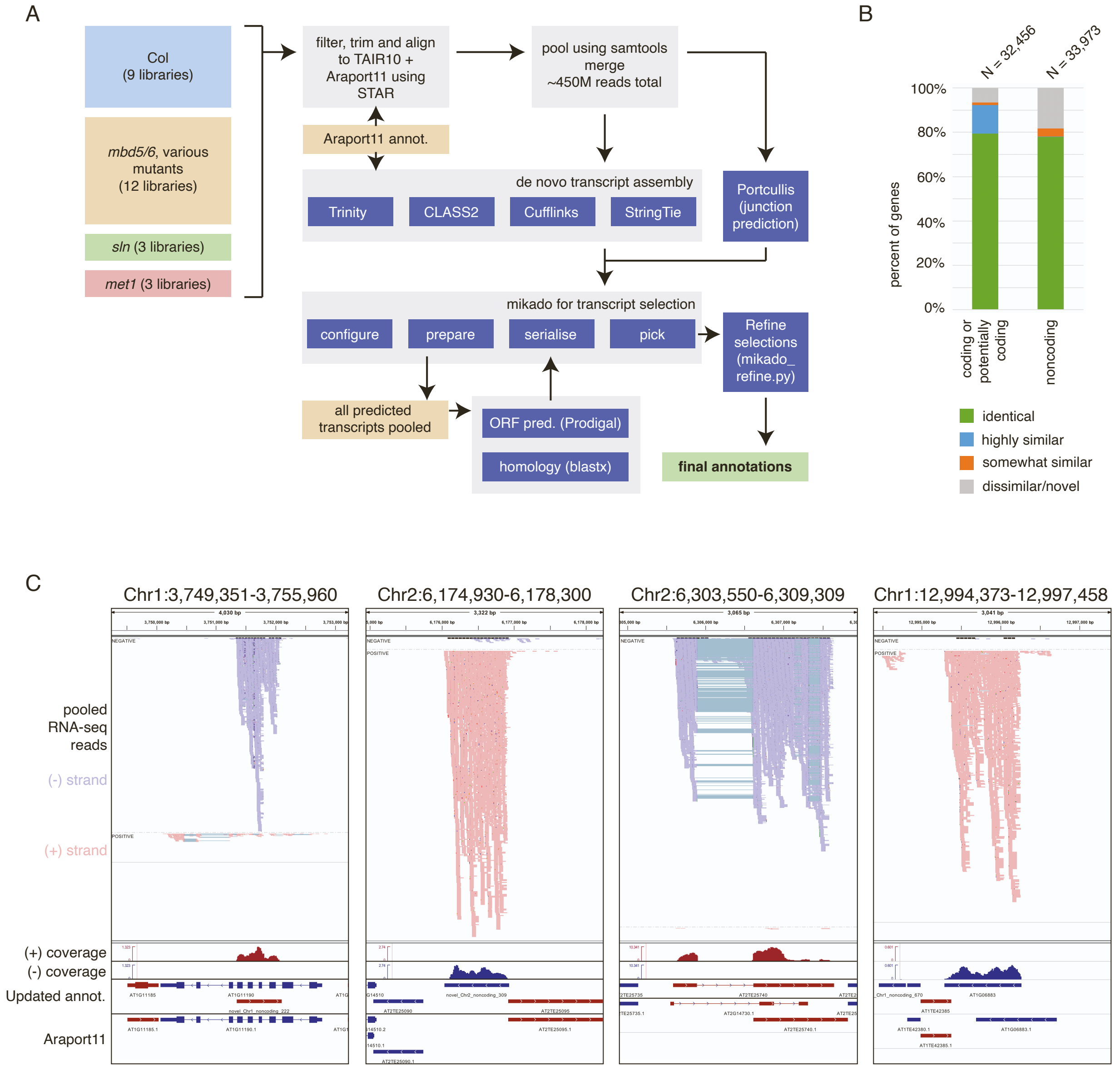

Figure S3 – related to Figure 2: Reannotation of *A. thaliana* transcriptome based on pollen data from *mbd5/6*, *sln*, and *met1*.

A) Overview of approach used to obtain reannotation, using a combination of four transcript assembly programs: Trinity v2.13.2 [S4], Cufflinks v.2.2.1 [S5], CLASS2 v.2.1.7 [S6] and StringTie v.2.1.6 [S7]. The program Mikado was used to select the best transcript. Transcript selection was further refined using a custom script (available on Github). B) Percentage of genes that were identical, highly similar, somewhat similar, and dissimilar/novel in the reannotation vs. in the original Araport11 annotations. Identical = all transcript features unchanged, highly similar (coding) = CDS unchanged but altered UTRs, highly similar (noncoding) = > 95% overlap of

exons, somewhat similar (coding) = CDS > 80% similar and in frame, somewhat similar (noncoding) = > 50% overlap of exons, all others considered dissimilar or novel. C) Example loci showing missing annotations added during reannotation (left two panels), and example loci where existing annotations were improved (right two panels). Top track shows reads from pooled BAM file, next two tracks indicate coverage originating from (+) or (-) strand. Top annotation track ("Updated annot." shows updated annotations while starting annotations from Araport11 are shown at bottom. Forward annotations are colored red, reverse colored blue. Note that reads mapping to (-) strand originate from (+) strand annotations and vice versa.

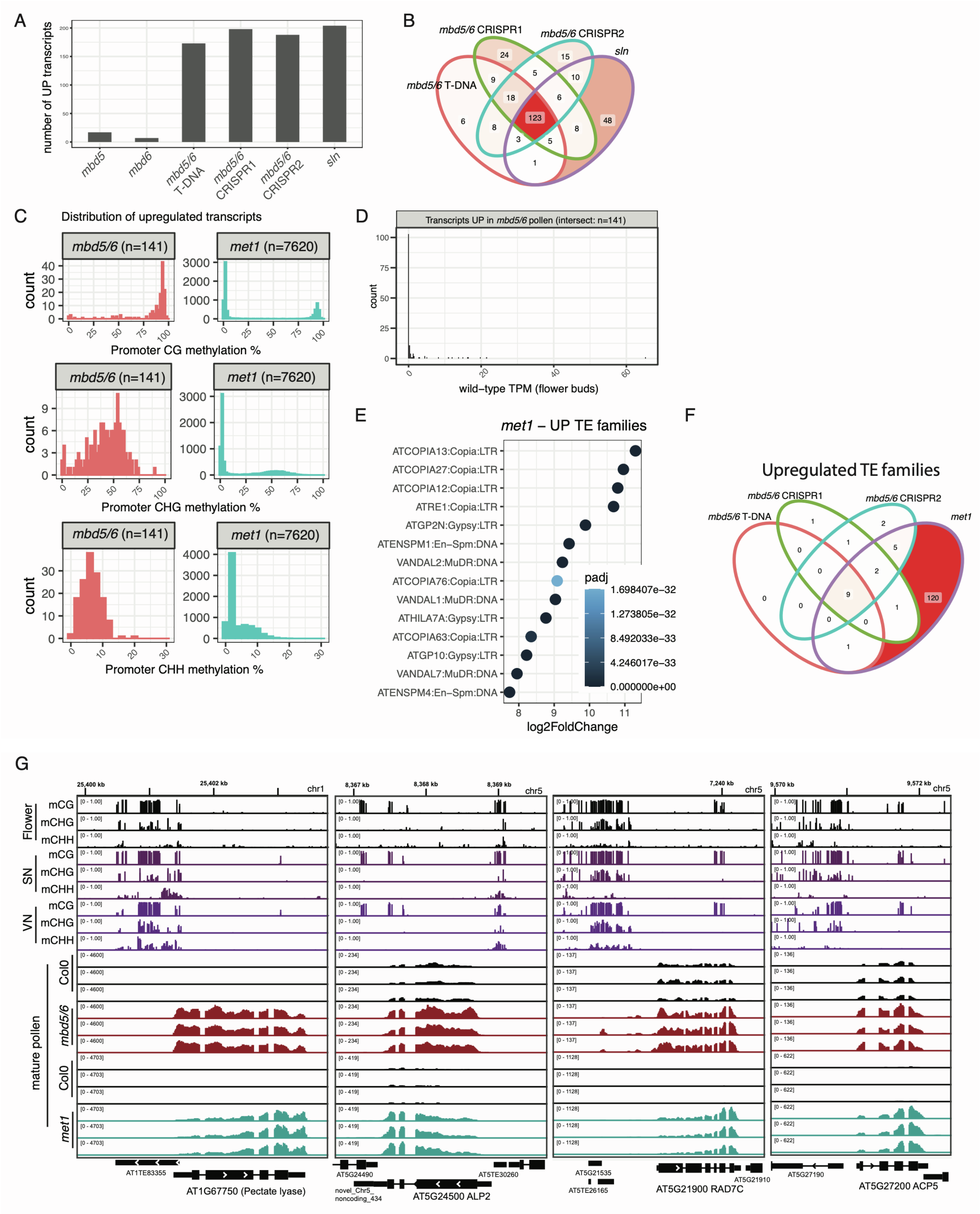

A
number of UP transcripts
mbd5
mbd6
mbd5/6 T-DNA
mbd5/6 CRISPR1
mbd5/6 CRISPR2
sln
B
mbd5/6 CRISPR1
mbd5/6 CRISPR2
sln
mbd5/6 T-DNA
24
15
9
5
10
18
6
6
123
48
8
8
3
5
1
C
Distribution of upregulated transcripts
mbd5/6 (n=141)
met1 (n=7620)
count
Promoter CG methylation %
Promoter CHG methylation %
Promoter CHH methylation %
D
Transcripts UP in mbd5/6 pollen (intersect: n=141)
count
wild−type TPM (flower buds)
E
met1 – UP TE families
ATCOPIA13:Copia:LTR
ATCOPIA27:Copia:LTR
ATCOPIA12:Copia:LTR
ATRE1:Copia:LTR
ATGP2N:Gypsy:LTR
ATENSPM1:En−Spm:DNA
VANDAL2:MuDR:DNA
ATCOPIA76:Copia:LTR
VANDAL1:MuDR:DNA
ATHILA7A:Gypsy:LTR
ATCOPIA63:Copia:LTR
ATGP10:Gypsy:LTR
VANDAL7:MuDR:DNA
ATENSPM4:En−Spm:DNA
padj
1.698407e−32
1.273805e−32
8.492033e−33
4.246017e−33
0.000000e+00
log2FoldChange
F
Upregulated TE families
mbd5/6 CRISPR1
mbd5/6 CRISPR2
mbd5/6 T-DNA
met1
120
G
Flower
SN
VN
mCG
mCHG
mCHH
mature pollen
Col0
mbd5/6
met1
chr1
chr5
AT1TE83355
AT1G67750 (Pectate lyase)
AT5G24490
AT5TE30260
novel_Chr5_noncoding_434
AT5G24500 ALP2
AT5G21535
AT5TE26165
AT5G21900 RAD7C
AT5G21910
AT5G27190
AT5G27200 ACP5

Figure S4 – related to Figure 2. Features of the MBD5/6 targets in mature pollen.

A) Number of upregulated transcripts detected by RNA-seq in mature pollen in the indicated mutants. B) Venn diagram showing the overlap between the upregulated transcripts in different mutants. C) Distribution of the promoters that are upregulated either in *mbd5/6* or in *met1* pollen based on their wild-type flower buds methylation levels in CG, CHG, or CHH context. D) Distribution of wild-type expression levels in flower buds for the *mbd5/6* upregulated transcripts. E) Analysis of upregulated TE families in *met1* (mature pollen). Only the top 14 TE families are displayed. F) Venn diagrams of overlaps between the mature pollen upregulated TE families in different mutants. G) Genome browser tracks of mature pollen RNA-seq in *mbd5/6* T-DNA and *met1-3*. Wild-type methylation tracks are shown as reference: flower data is from [S3], vegetative nucleus (VN) and sperm nucleus (SN) data is from [S8].

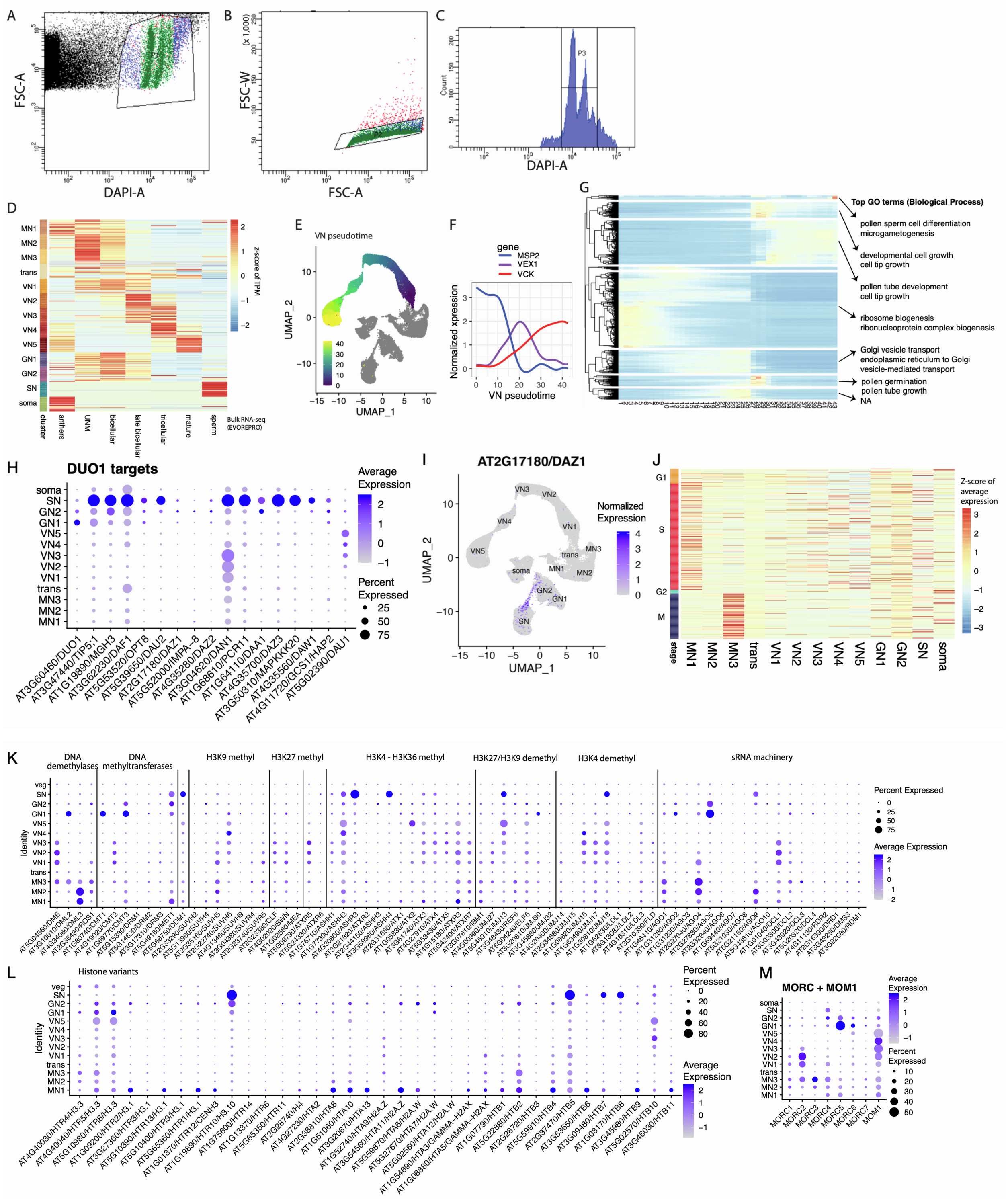

Figure S5 – related to Figure 3. Characterization of snRNA-seq clusters.

A,B,C) Representative plots showing the parameters used to isolate and purify the pollen nuclei for snRNA-seq. The gates in panel A were used to separate the DAPI positive nuclei from the small debris, the gates in panel B were used to remove nuclei aggregates (red). The P3 population (Panel C) was sorted in a single tube and used for snRNA-seq. The two peaks likely correspond to the large and small nuclei (VN and SN/GN) as the different chromatin structure can lead to a different staining intensities. D) Heatmap of Col0 bulk RNA-seq datasets downloaded from https://evorepro.sbs.ntu.edu.sg/ [S9]. Rows correspond to the top 20 markers for each cluster (ranked by average log2FC) of the snRNA-seq data from this study (the list of markers is available in Table S3). E) UMAP displaying all the wild-type nuclei with their assigned pseudotimes in the VN developmental trajectory (color code). F) Expression pattern along the VN pseudotime for an early VN (*MSP2*), a mid VN (*VEX1*) and a late VN (*VCK*) gene. The lines represent a smoothed trend (ggplot *geom_smooth*) of the log-normalized expression of each gene. G) Heatmap showing 3,135 genes that change as a function of pseudotime (see Methods). The genes were split into 8 groups based on hierarchical clustering. The top ranked GO term for each group is shown (full list in Table S5). H) Dotplot of cluster specific expression profiles for DUO1 and a list of genes that were previously annotated as DUO1 targets [S10]. The dot size represents the percentage of cells in which the gene was detected, the dot color represents the scaled average expression for each cluster. I) UMAP showing the expression pattern of DAZ1, which is specifically expressed in the transition between GN2 and SN. J) Heatmap showing the scaled average expression pattern across all snRNA-seq clusters for a list of previously published cell-cycle marker genes [S11]. Only the genes with a standard deviation higher than 0.2 across all clusters were displayed (n=405). The MN1 and MN3 clusters show a clear enrichment for S-phase and M-phase markers respectively. K,L,M) Dotplot of cluster specific expression profiles for the indicated groups of genes (see panel H).

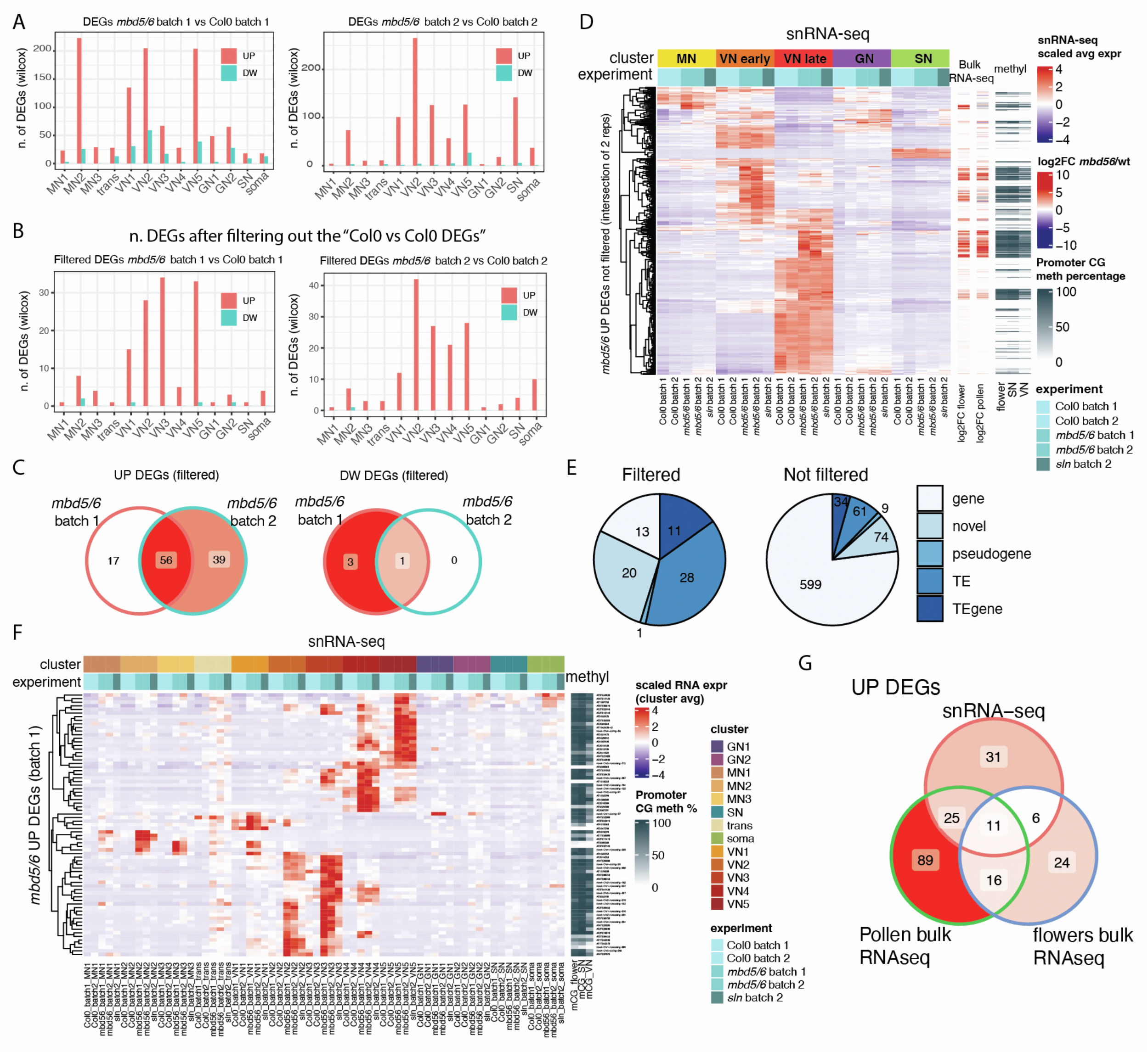

Figure S6 – related to Figure 4. Blacklisting the high variability genes allows detection of high-confidence DEGs with snRNA-seq data.

A,B) Barplots of the *mbd5/6* DEGs obtained without filtering (A) or after filtering out the DEGs obtained when comparing Col0 samples to each other (B) (see Methods for details). C) Overlaps of upregulated or downregulated DEGs in two independent experiments. D) Heatmap representation of the not-filtered *mbd5/6* upregulated genes (intersection between batch 1 and batch 2). Shown is the snRNA-seq scaled expression level of the cluster averages in the indicated samples. For each gene, the log2 fold-change obtained by bulk RNA-seq in flower buds or pollen is shown on the right. The last three columns on the right indicate the wild-type CG methylation

percentage at the promoters of each gene (600 bp windows centered at the TSS). The VN and SN BS-seq data is from [S8]. The genes with a strong positive fold-change in bulk RNA-seq tend to be promoter methylated. E) Classification of the *mbd5/6* upregulated DEGs obtained by snRNA-seq (union of all clusters) either with or without filtering. The "novel" genes were identified via a transcript reannotation based on all the mature pollen RNA-seq datasets included in this study (see Methods and Figure S3). F) Heatmap representation of the union of the *mbd5/6* upregulated genes obtained in each cluster (filtered). Shown is the snRNA-seq scaled expression level of the cluster averages in the indicated samples. The methylation data is the same as in panel D. G) Venn diagram of overlaps between snRNA-seq batch 1 and bulk-RNAseq upregulated DEGs.

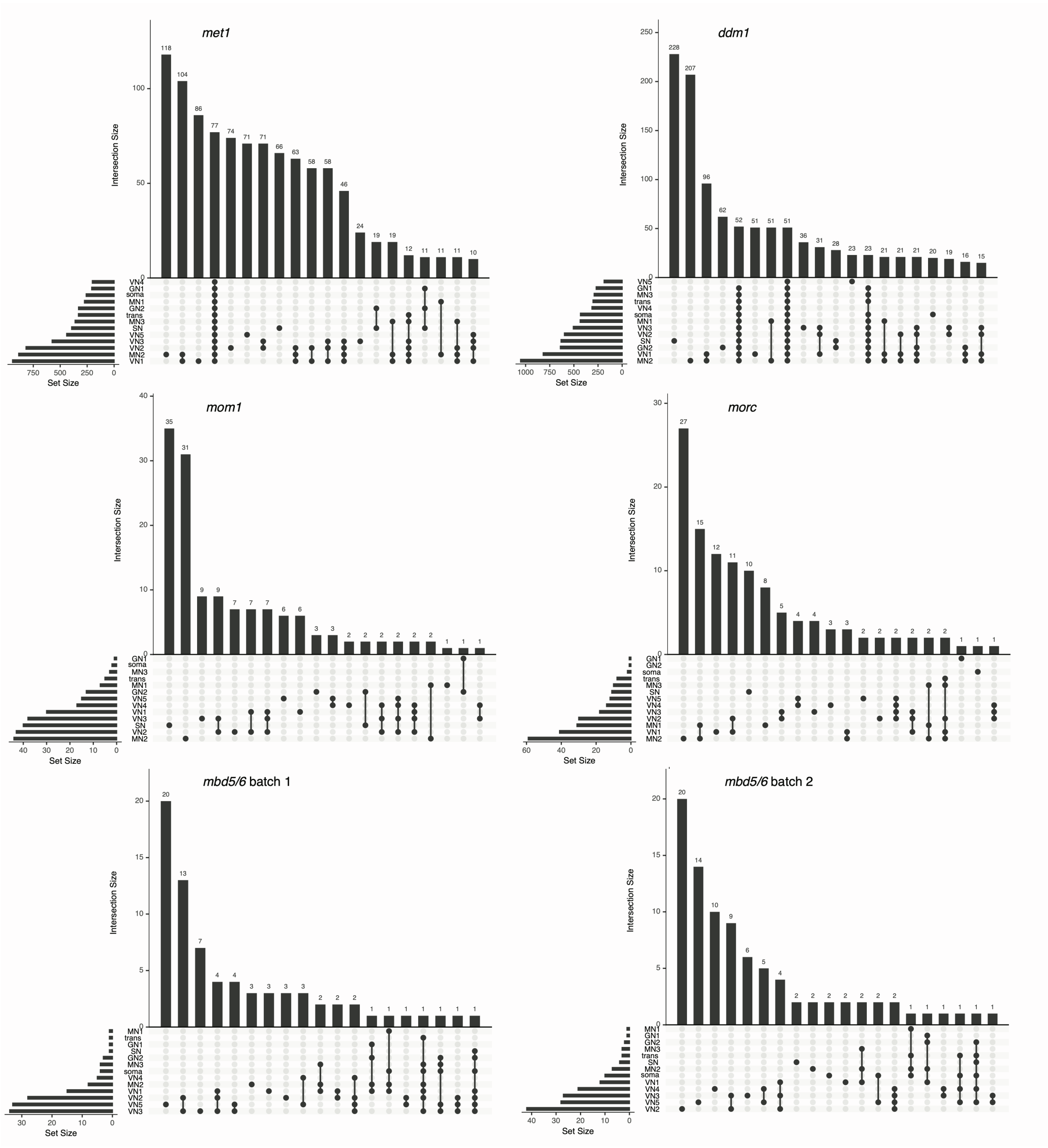

Figure S7– related to Figure 5: Differential gene expression analysis at individual nuclei clusters.

Upset plots showing the intersection of the upregulated transcripts obtained in each nucleus type, for the indicated genotypes.

Supplementary references

1. Kurihara, D., Mizuta, Y., Sato, Y., and Higashiyama, T. (2015). ClearSee: A rapid optical clearing reagent for whole-plant fluorescence imaging. Development *142*, 4168–4179. 10.1242/dev.127613.
2. Stroud, H., Hale, C.J., Feng, S., and Jacob, C.E. (2012). DNA Methyltransferases Are Required to Induce Heterochromatic Re-Replication in Arabidopsis. PLoS Genet *8*, e1002808. 10.1371/journal.pgen.1002808.
3. Ichino, L., Boone, B.A., Strauskulage, L., Harris, C.J., Kaur, G., Gladstone, M.A., Tan, M., Feng, S., Jami-Alahmadi, Y., Duttke, S.H., et al. (2021). MBD5 and MBD6 couple DNA methylation to gene silencing through the J-domain protein SILENZIO. Science *372*, 1434–1439. 10.1126/science.abg6130.
4. Haas, B.J., Papanicolaou, A., Yassour, M., Grabherr, M., Blood, P.D., Bowden, J., Couger, M.B., Eccles, D., Li, B., Lieber, M., et al. (2013). De novo transcript sequence reconstruction from RNA-seq using the Trinity platform for reference generation and analysis. Nat. Protoc. *8*, 1494–1512. 10.1038/NPROT.2013.084.
5. Trapnell, C., Williams, B.A., Pertea, G., Mortazavi, A., Kwan, G., Van Baren, M.J., Salzberg, S.L., Wold, B.J., and Pachter, L. (2010). Transcript assembly and quantification by RNA-Seq reveals unannotated transcripts and isoform switching during cell differentiation. Nat. Biotechnol. *28*, 511–515. 10.1038/NBT.1621.
6. Song, L., Sabunciyan, S., and Florea, L. (2016). CLASS2: accurate and efficient splice variant annotation from RNA-seq reads. Nucleic Acids Res. *44*. 10.1093/NAR/GKW158.
7. Pertea, M., Pertea, G.M., Antonescu, C.M., Chang, T.C., Mendell, J.T., and Salzberg, S.L. (2015). StringTie enables improved reconstruction of a transcriptome from RNA-seq reads. Nat. Biotechnol. *33*, 290–295. 10.1038/NBT.3122.
8. Ibarra, C.A., Feng, X., Schoft, V.K., Hsieh, T.F., Uzawa, R., Rodrigues, J.A., Zemach, A., Chumak, N., Machlicova, A., Nishimura, T., et al. (2012). Active DNA Demethylation in Plant Companion Cells Reinforces Transposon Methylation in Gametes. Science *337*, 1360–1364. 10.1126/science.1224839.
9. Julca, I., Ferrari, C., Flores-Tornero, M., Proost, S., Lindner, A.-C., Hackenberg, D., Steinbachová, L., Michaelidis, C., Pereira, S.G., Misra, C.S., et al. (2021). Comparative transcriptomic analysis reveals conserved programmes underpinning organogenesis and reproduction in land plants. Nat. Plants 2021 *7*, 1143–1159. 10.1038/s41477-021-00958-2.

10. Borg, M., Brownfield, L., Khatab, H., Sidorova, A., Lingaya, M., and Twell, D. (2011). The R2R3 MYB transcription factor DUO1 activates a male germline-specific regulon essential for sperm cell differentiation in Arabidopsis. Plant Cell *23*, 534–549. 10.1105/tpc.110.081059.
11. Menges, M., Hennig, L., Gruissem, W., and Murray, J.A.H. (2003). Genome-wide gene expression in an Arabidopsis cell suspension. Plant Mol. Biol. *53*, 423–442. 10.1023/B:PLAN.0000019059.56489.ca.
